# miR-191 affects skeletal muscle differentiation by regulating *Wwp1* in mouse myoblasts

**DOI:** 10.1371/journal.pone.0355370

**Published:** 2026-09-09

**Authors:** Wenhua Pu, Meimei Zhang, Siman Guo, Xiang Bao, Wenjia Cui, Chuncheng Liu

**Affiliations:** 1 Inner Mongolia Key Laboratory of Life Health and Bioinformatics, School of Life Science and Technology, Inner Mongolia University of Science & Technology, Baotou, China; 2 Department of Geriatrics, Ordos Central Hospital, Ordos, Inner Mongolia, China; Rosalind Franklin University of Medicine and Science Chicago Medical School, UNITED STATES OF AMERICA

## Abstract

Skeletal muscle atrophy is a key complication of various diseases, such as chronic obstructive pulmonary disease (COPD) and cancer. The mechanisms by which these diseases affect skeletal muscle metabolism need to be deeply explored. By analyzing the miRNA expression profiles in the plasma of patients with COPD, we found that miR-191 expression was significantly altered and it may influence skeletal muscle metabolism by regulating ubiquitination and the mTOR pathway. Using a mouse model of skeletal muscle injury induced by cardiotoxin, we found that miR-191 and *Wwp1* showed a dynamic negative correlation in injury repair. Transfection with miR-191 mimics significantly inhibited the expression of myogenic regulatory factor Myog and differentiation markers Myh1/7/8, while downregulating key genes in the mTOR pathway. Molecular mechanism studies showed that miR-191 could directly act on the 3’ untranslated region of the *Wwp1* gene to inhibit its expression. This study reveals the important role of the miR-191/Wwp1 axis in skeletal muscle differentiation and provides a novel theoretical basis for research on muscle atrophy induced by COPD, cancer cachexia, and other diseases.

## Introduction

In the human body, skeletal muscle is typically the heaviest organ system and plays a crucial role in supporting and protecting the body, enabling movement, as well as regulating protein synthesis and degradation. The inhibition of protein synthesis and the activation of protein degradation both lead to skeletal muscle atrophy. Skeletal muscle atrophy refers to the loss of muscle mass or fiber size, accompanied by the impairment or loss of associated physical functions such as walking speed or grip strength [1]. Since skeletal muscle atrophy is closely related to metabolism, changes in multiple tissues and organs under both physiologically and pathologically (such as in chronic obstructive pulmonary disease (COPD), cancer, and renal injury), can lead to muscle atrophy [2]. Muscle atrophy can impact quality of life and even cause patient death due to respiratory failure. However, as a complication, skeletal muscle atrophy often manifests with subtle symptoms during the early stages of disease progression, making it easy to overlook in clinical practice [3].

Muscle atrophy mainly results from an imbalance between protein breakdown and synthesis in myofibers, reducing muscle regeneration potential [4]. A group of genes termed atrogenes shows significant expression changes in various skeletal muscle atrophy conditions. Some of these atrophy-related genes directly belong to the two major skeletal muscle degradation systems: the ubiquitin-proteasome system (UPS) and autophagy-lysosome pathway (ALP) [5]. E3 ubiquitin ligases are key components of the ubiquitin-proteasome system [6]. Among atrogenes, Atrogin-1 and MuRF-1 belong to the E3 ubiquitin ligase family and serve as crucial regulators of ubiquitin-mediated protein degradation in skeletal muscle [7]. miRNAs play a significant role in skeletal muscle atrophy by influencing the ubiquitin-proteasome system. For instance, miR-29 can resist muscle atrophy by inhibiting the expression of MuRF1 [8] or promote skeletal muscle hypertrophy by regulating Mstn [9], while miR-18a can cause myotube atrophy by suppressing the IGF-1/Akt pathway and activating the ubiquitin-proteasome system [10].

Extramuscular factors can also contribute to skeletal muscle atrophy by influencing muscle atrophy-related elements. For instance, extracellular vesicles deliver heat shock proteins (HSP70 and HSP90) to myofibers, which modulate the expression of E3 ubiquitin ligases, thereby inducing skeletal muscle atrophy [11]. Inflammatory factors associated with other diseases can directly bind to their receptors and activate a series of downstream signaling pathways, including the NF-κB, JAK/STAT, and p38-MAPK pathways. This process inhibits muscle protein synthesis, overactivates proteolysis, and ultimately leads to skeletal muscle atrophy [2]. In addition, skeletal muscle atrophy can also be caused by muscle stem cell abnormalities [12].

COPD, cachexia, and renal injury are among the various disorders that can lead to skeletal muscle atrophy. COPD, a chronic inflammatory lung disease, often leads to diaphragm dysfunction. Multiple studies have demonstrated that skeletal muscle dysfunction in COPD patients correlates with more severe airflow obstruction, pulmonary emphysema, and increased mortality [13,14]. Notably, IL-6 is highly expressed in COPD patients and recognized as a biomarker of systemic inflammation. In addition to inducing inflammation, chronic hypoxia may also contribute to muscle mass loss [15,16]. Cachexia, characterized by an involuntary loss of muscle and fat tissue, is a complex wasting syndrome. It is defined by systemic inflammatory responses, elevated pro-inflammatory cytokines (IL-1, IL-6, and TNF-α), and significant impacts on both IGF-1/Akt and NF-κB pathways [17]. Renal injury induces skeletal muscle atrophy through mechanisms involving inflammation-cytokine imbalance, impaired muscle stem cell function, accumulation of uremic toxins, and metabolic acidosis [18].

As crucial cargoes of extracellular vesicles, miRNAs play regulatory roles in skeletal muscle atrophy associated with COPD, cachexia, and renal injury; however, these roles require further investigation. We analyzed differentially expressed miRNAs in the serum of COPD patients and identified a significant alteration in the expression of miR-191. Functional studies in myoblast C2C12 cells revealed that miR-191 suppressed the expression of its target gene *Wwp1*, modulated key genes in the mTOR pathway, and inhibited skeletal muscle differentiation. This study provides a theoretical basis for understanding muscle wasting associated with conditions such as COPD.

## Materials and Methods

### Injection of cardiotoxin (CTX)

Ten-week-old C57BL/6 mice were utilized to establish a cardiotoxin-induced skeletal muscle injury model. Mice were housed in an individually ventilated cage (IVC) system, with the temperature maintained between 22–26 °C and relative humidity between 45%−65%. Mice were maintained under a 12-hour light/12-hour dark cycle with ad libitum access to chow and water during the experiment. For cardiotoxin injection, mice were anesthetized via intraperitoneal administration of pentobarbital sodium (50 mg/kg). The fur overlying the tibialis anterior (TA) muscle was shaved, and the injection site was disinfected with an alcohol swab. Using a microsyringe, 100 μL of CTX solution (10 μM in PBS) was slowly injected into the tibialis anterior muscle. After injection, the mice were placed on a heating table until recovery from anesthesia and subsequently returned to their cages. Mice were randomly assigned to three groups: PBS‑injected control (n = 5, harvested on day 3), CTX‑injected day 1 group (n = 3, harvested on day 1), and CTX‑injected day 3 group (n = 3, harvested on day 3). At the indicated endpoints, mice were euthanized by cervical dislocation. No analgesics were used to avoid interference with muscle regeneration; mice were monitored twice daily and no severe pain was observed. The animal protocol was approved by the Institutional Animal Care and Use Committee of Inner Mongolia University of Science and Technology (approval No. NMGKJDX‑2022‑9‑22).

### Cell culture and differentiation

#### Cell culture.

C2C12 and HEK293T cells were preserved in our laboratory. Cells were cultured in a humidified incubator at 37°C with 5% CO_2_. The growth medium used during cell culture was DMEM supplemented with 10% fetal bovine serum (FBS). During the differentiation of C2C12 cells, the medium was switched to DMEM supplemented with 2% horse serum. Both media were supplemented with 1% penicillin-streptomycin (PS).

#### Induction of myoblast differentiation.

When C2C12 cells reached 95% confluence in the culture dish, differentiation was induced. The growth medium was removed without rinsing with PBS, and differentiation medium was directly added. For each well of a 6-well plate, 4.5 mL of differentiation medium was added. Unless transfection was performed, the medium was not replaced throughout the differentiation period.

### Construction of psiCHECK-2 vector

The modification process of psiCHECK-2 vector (Promega (Beijing) Biotech Co., Beijing, China) was performed as previously described [19]. The basic procedure was as follows: First, the 3'UTR of the *Wwp1* gene (mouse) containing the miR-191 binding site was amplified using primers WT-F and WT-R. Then, the PCR product was ligated into the multiple cloning site in the psiCHECK-2 vector to obtain the psi-wt-Wwp1 vector.

The mutation of the miR-191 binding site was achieved through overlap extension PCR. Fragment 1 was amplified using primers WT-F and mut-R with psi-wt-Wwp1 as the template. Fragment 2 was amplified using primers mut-F and WT-R with psi-wt-Wwp1 as the template. Then, the mutated template was obtained using fragment 1 and fragment 2 as templates and primers. Subsequently, the mutated full-length fragment was amplified using primers WT-F and WT-R, and cloned into the psiCHECK-2 vector to obtain psi-m-Wwp1.

The primer sequences are as follows:

WT-F: 5’-CCGCTCGAGTGCATTTAAATACCCAGCCAAGA-3’
WT-R: 5’-ATAAGAATGCGGCCGCTGTTTAATACACCTGGCGCTC-3’
mut-F: 5’-TATAAATGTTTTCCGTTCTTCCACAGA-3’
mut-R: 5’-TCTGTGGAAGTGATCGTAACATTTATA-3’

### Transfection

miRNA mimics, siRNA, or vectors were transfected into cells using liposomes (Lipo6000™, C0526, Beyotime, China). The sequence of miR-191 mimics is CAACGGAAUCCCAAAAGCAGCUG. The catalog number of NC is miR1N0000002-1–5. The catalog number of siWwp1 is siB12329162409-1–5. The catalog number of siNC is siN0000001-1–5. All of the above nucleic acids were synthesized by RiboBio Co., Ltd. (Guangzhou, China). The transfection procedure was performed as previously described [20] and briefly summarized below:

#### Transfection protocol for myoblasts.

First, the appropriate amount of miR-191 mimics or siRNA (240 pmol/well for proliferating phase transfection, 320 pmol/well for pre-differentiation transfection, and 600 pmol/well for differentiation phase transfection) was mixed with 250 μL of serum-free medium (Opti-MEM™, 51985034, Gibco) in a sterile centrifuge tube. In another sterile centrifuge tube, the appropriate amount of transfection reagent (1 µL of transfection reagent per 40 pmol of mimics) was mixed with 250 μL of serum-free medium. The two solutions (one containing nucleic acid and the other containing the transfection reagent) were gently mixed and incubated at room temperature for 20 min.

During this incubation period, the old medium was removed from the cells and 1 mL of fresh serum-free medium was added. After the incubation, the prepared transfection complexes were added dropwise to the cells in a 6-well plate, and the culture plate was gently shaken to ensure even distribution of the complexes. The culture plate was placed back in the incubator, and after 5 h of incubation, the medium containing the transfection complexes was aspirated and fresh medium (growth medium or differentiation medium, depending on the experimental requirements) was added to continue cell culture.

#### Transfection protocol for HEK293T cell.

First, 40 pmol of miR-191 mimics and 500 ng of psiCHECK-2 vector (psi-wt-Wwp1 or psi-m-Wwp1) were mixed with 75 μL of serum-free medium in a sterile centrifuge tube. In another sterile centrifuge tube, 1.5 μL transfection reagent was mixed with 75 μL of serum-free medium. The serum-free medium containing nucleic acid and that with the transfection reagent were gently mixed and incubated at room temperature for 20 min.

During this incubation period, remove the old medium from the cells and add 200 μL of fresh serum-free medium. After the incubation, the prepared transfection complexes were added dropwise to the cells in a 24-well plate, and the culture plate was gently shaken to ensure even distribution of the complexes.

The culture plate was placed back in the incubator, and after 5 h of transfection, without aspirating the medium containing the transfection complexes, 600 μL of fresh medium was directly added, and the cells were cultured for an additional 19 h. 24 h after transfection, the activities of Renilla and Firefly luciferases were measured using the Dual-Luciferase® kit (Promega (Beijing) Biotech Co., China). Cells were harvested and fully lysed using 1 × Passive Lysis Buffer (PLB) from the Dual-Luciferase® kit. Firefly luciferase activity was measured by adding Luciferase Assay Reagent II (LARII), and the luminescence was immediately read using the luminometer. Subsequently, Stop & Glo® Reagent was added to the same well to quench the firefly signal and simultaneously initiate the Renilla luciferase reaction, and the second reading was taken. The ratio of Renilla to Firefly luciferase activity was calculated for each well to normalize for transfection efficiency and cell number.

### Quantitative reverse transcription polymerase chain reaction (RT-qPCR)

#### RNA extraction.

Cells or homogenized tissues were thoroughly lysed using Trizol reagent (Takara Bio, Japan). The cell samples refer to the myoblast cell line C2C12 used in the experiments shown in Figs 3–6; the tissue samples refer to the tibialis anterior muscle from the CTX-injected mouse experiment shown in Fig 2C–D. Phase separation of aqueous (RNA), protein and organic components was achieved by chloroform treatment. RNA was precipitated with isopropanol and washed with 75% ethanol to remove impurities. The RNA pellet was finally dissolved in 20 μL RNase-free water.

#### Reverse transcription (cDNA synthesis).

Reverse transcription of mRNA or miRNA was performed using oligo(dT) primers or stem-loop specific primers respectively. Complementary DNA (cDNA) was synthesized using M-MLV reverse transcriptase (Promega, WI, USA), dNTPs, and RNase inhibitor with mRNA/miRNA as template. The stem-loop primer for miR-191-5p was as follows:

miR-191-5p-RT:

5’-CTCAACTGGTGTCGTGGAGTCGGCAATTCAGTTGAGCAGCTGCT-3’

#### Real-time quantitative PCR (qPCR).

The cDNA generated from RNA reverse transcription was detected using SYBR Green reagent (Beijing Zoman Biotechnology Co., Ltd, China). The instrument used was ABI 7500. *Gapdh* or U6 were used as internal references. The U6 reverse transcription and qPCR primers were referred to in the published manuscript [21]. Based on the cycle number (Ct value) when the fluorescence signal reached the threshold, the expression differences of miR-191 or the target gene were calculated (2^-ΔΔCt^).

The qPCR primers are listed in Supplementary Table in S1 File.

### Western blot

RIPA lysis buffer (containing 1% PMSF) was added to the cells in 6-well plates, followed by incubation on ice for 30 min. The lysate was transferred to centrifuge tubes and centrifuged at 12000 rpm for 15 min at 4°C. The supernatant was collected as the extracted protein sample.

Protein concentration was quantified using the BCA method. Briefly, a standard curve was created, samples were diluted, BCA reagent was added, and the mixture was incubated at 37°C for 30 min. Absorbance was measured at 562 nm with a microplate reader, and protein concentration was calculated using the blank control and standard curve.

Protein samples were separated by SDS-PAGE and transferred to PVDF membranes for subsequent hybridization. After transfer, membranes were blocked with 5% skim milk (prepared in TBST) at room temperature for 1 h with shaking. Primary antibodies, including MyHC (1:5000; Sigma, MO, USA), p-AKT (Ser473) (1:2000; CST, MA, USA), AKT (1:2000; CST, MA, USA), mTOR (1:2000; CST, MA, USA) and Gapdh (1:5000; CST, MA, USA), were applied and incubated at 4°C overnight. HRP-conjugated goat anti-mouse/anti-rabbit secondary antibody (1:10000; ZSBio, China) was then added and incubated at room temperature with shaking for 1 h. Protein bands were visualized using an ECL reagent (Beyotime, China), and signals were captured using a gel imaging system.

### Cell cycle flow cytometry

24 h after transfection, cells were digested with trypsin, collected by centrifugation, and washed with PBS. Cells were fixed with 70% ethanol at 4°C overnight. After fixation, cells were washed again with PBS and stained with propidium iodide (PI) at a concentration of 50 μg/mL [22]. Following 30 min incubation at 4°C and additional PBS washing, cell cycle analysis was performed using FACSCalibur flow cytometer (Becton Dickinson, NJ, USA). A total of 20000 cells were counted per sample, and data were analyzed using ModFit LT cell cycle analysis software.

### Analysis of single-nucleus RNA-seq data from a mouse model of CTX-induced skeletal muscle injury (GSE272412)

We analyzed a publicly available single-nucleus RNA-seq dataset (GSE272412), in which mice were subjected to CTX-induced injury in the tibialis anterior muscle and sampled 3 days post-injury. From the expression matrix, we extracted the myocyte subpopulation. For each age group following CTX injury, we calculated the proportion of Wwp1-positive myocytes and the average expression level of Wwp1 within the myocyte population.

### Statistical analysis

The target genes of miR-191 were predicted using the databases miRDB, ENCORI, and TargetScan 8.0. The 3'UTR sequences of the *Wwp1* gene from humans, chimpanzees, rhesus monkeys, cattle, dogs, rats, mice, opossums, and chickens were analyzed using MEGA 11 (Neighbor-Joining method), and a phylogenetic tree was constructed [23]. Gene clustering analysis was performed through the clusterProfiler package of the R language. Protein-Protein Interaction (PPI) analysis was performed using the STRING database [24] as described in the published manuscript [25].

All experiments were performed with at least three independent biological replicates unless otherwise specified. The exact number of biological replicates (n) for each experiment is indicated in the corresponding figure legends. Experimental results were presented as the mean ± SEM. Statistical significance was calculated by Student’s t-test. SPSS 16.0 was used for Student’s t-test in this experiment. *p* < 0.05 was considered statistically significant. *: *p* < 0.05; **: *p* < 0.01.

## Results

### miR-191 and *Wwp1* gene are associated with skeletal muscle

**Fig 1:**
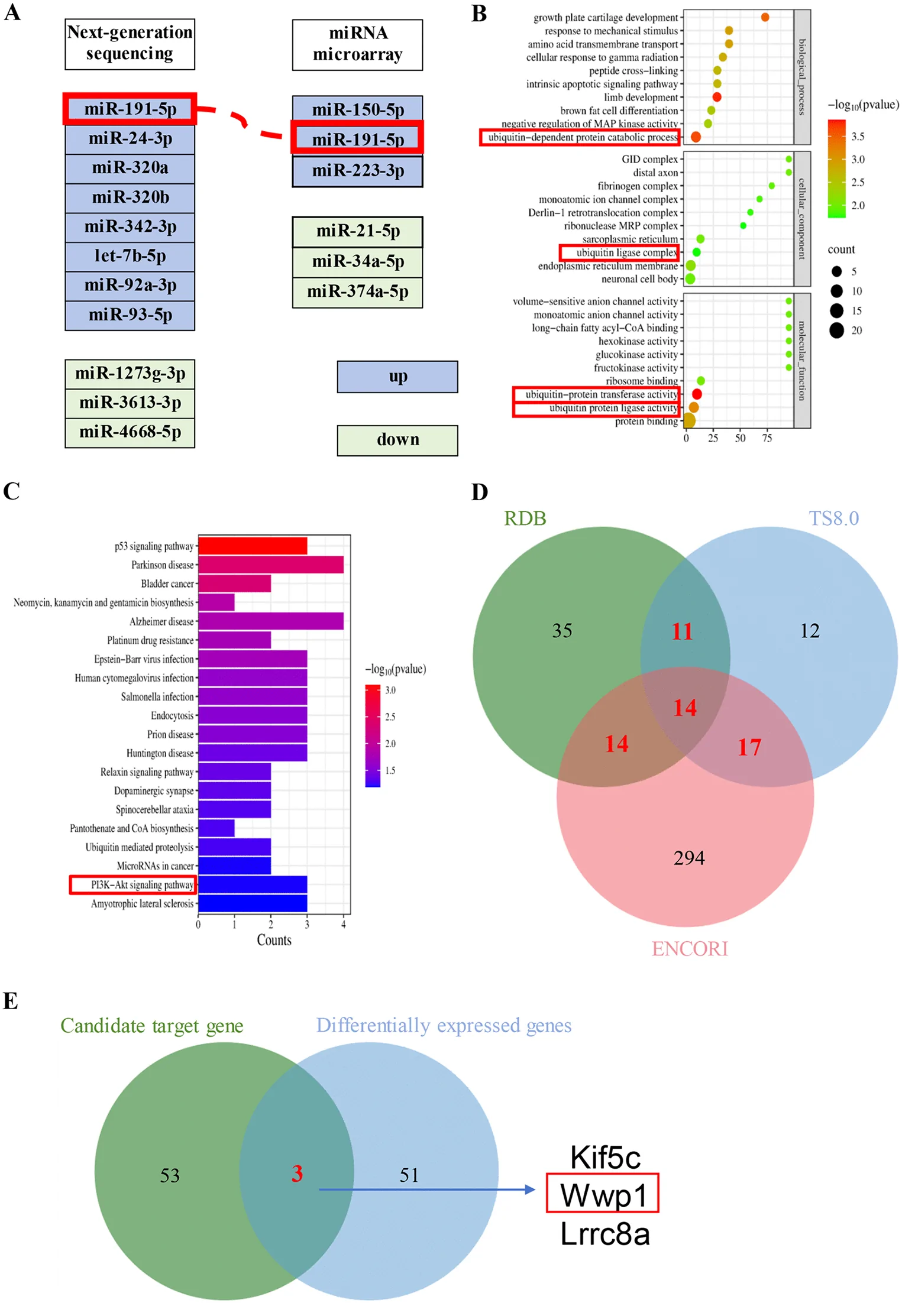
Screening of candidate target genes regulated by miR-191. (A) Analysis and screening of differentially expressed miRNAs in the serum of patients with chronic obstructive pulmonary disease. (B) GO enrichment analysis of differentially expressed genes induced by miR-191-5p overexpression (Top10 for each ontology). The y-axis shows GO terms, and the x-axis represents enrichment score, and the color represents -log_10_^(p-value)^. (C) KEGG enrichment analysis of differentially expressed genes induced by miR-191-5p overexpression (Top20). The y-axis indicates KEGG pathways, the x-axis shows the number of differentially expressed genes enriched in each pathway, and the color represents -log_10_^(p-value)^. (D) Prediction of target genes of miR-191-5p using RDB, TargetScan Mouse 8.0 (TS8.0), and ENCORI databases, and identification of candidate target genes through Venn diagram analysis. (E) Venn diagram analysis of overlapping differentially expressed genes and candidate target genes.

To explore the potential role of miR-191 in skeletal muscle, we first re-analyzed publicly available miRNA expression datasets from COPD patients. As shown in Fig 1A, analysis of serum exosomal miRNA-seq data from Sundar et al. [26] and serum miRNA microarray data from Velasco-Torres et al. [27] revealed that miR-191-5p expression was consistently altered in both studies. To further investigate the biological processes that miR-191 may regulate, we re-analyzed the expression changes of miR-191-5p in the serum of tumor-bearing mice [28] or cancer patients [29], as well as in urine samples collected after kidney injury [30,31], and found that miR-191 expression levels were significantly altered under all of the above pathological conditions (Figure S1 in S1 File).

We re-analyzed the RNA-seq data from HeLa cells transfected with miR-191, as published by Damon Polioudakis et al [32]. Differentially expressed genes were identified using a threshold of p < 0.05 and a fold change (FC) ≥ 1.5. Based on these differentially expressed genes, GO and KEGG pathway enrichment analyses were performed. The GO enrichment results suggested that miR-191 may be involved in ubiquitination processes, which are critical for skeletal muscle cell proliferation, differentiation, and homeostasis. Meanwhile, KEGG analysis highlighted the involvement of miR-191 in the PI3K signaling pathway, a key regulator of skeletal muscle metabolism, hypertrophy, and atrophy.

To determine if miR-191-5p might regulate genes related to the above-mentioned processes, we used three miRNA related databases—RDB, TargetScan Mouse 8.0, and ENCORI—to predict target genes of miR-191-5p. Candidate target genes were defined as those predicted in at least two databases. Venn diagram analysis identified 56 candidate target genes (Fig 1D). Subsequently, we conducted a joint analysis of differentially expressed genes and candidate target genes, revealing that *Kif5c*, *Wwp1*, and *Lrrc8a* were both differentially expressed genes and candidate targets of miR-191-5p (Fig 1E).

**Fig 2:**
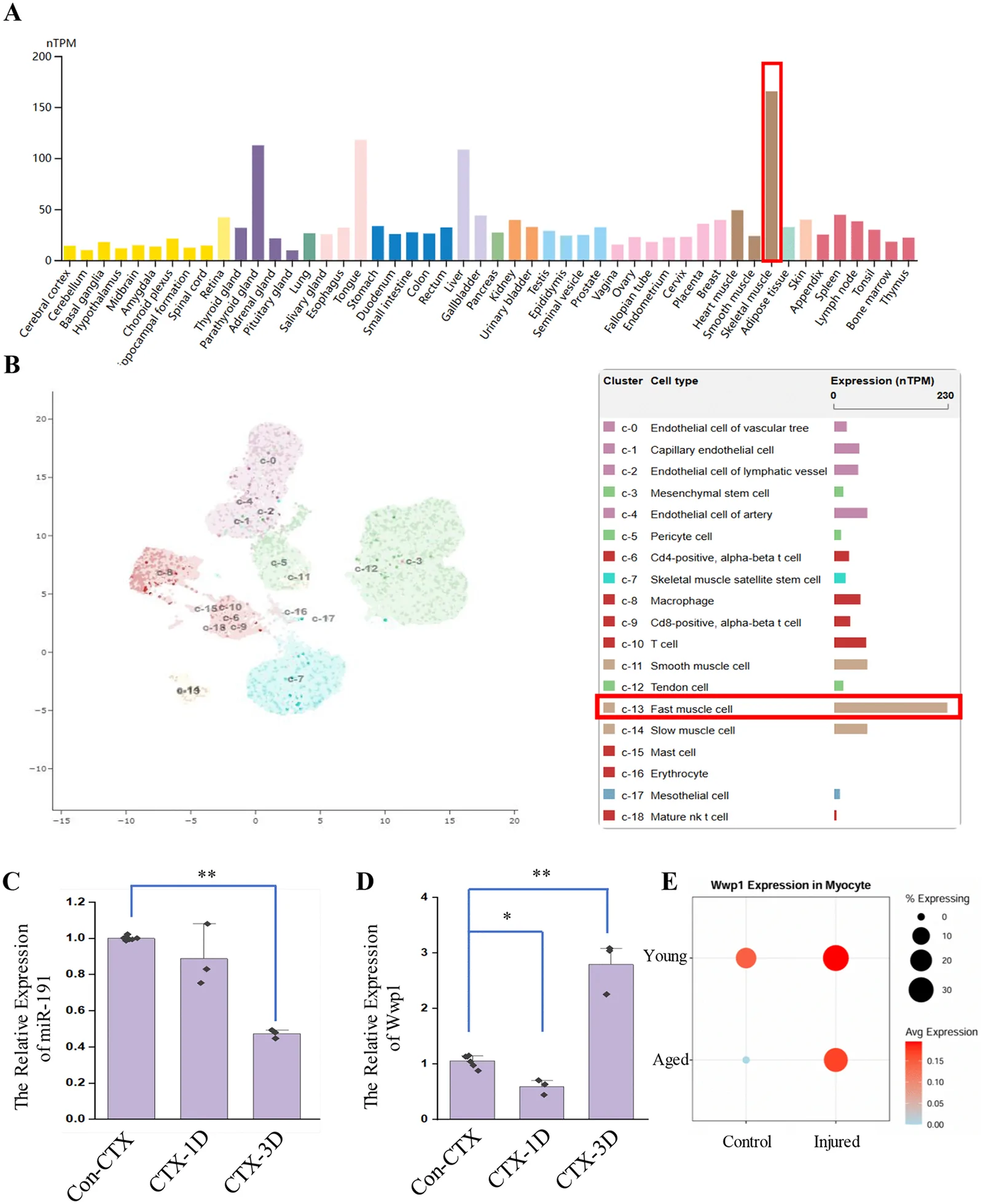
Expression of *Wwp1* gene in skeletal muscle. (A) Expression of *WWP1* gene across human tissues (databases: The Human Protein Atlas). (B) Analysis of *WWP1* gene expression in different skeletal muscle cell types based on single-cell sequencing of skeletal muscle. (C)-(D) RT-qPCR was used to verify the expression of miR-191 and *Wwp1* at 1 d and 3 d after CTX or PBS injection. n = 3 (CTX injection), n = 5 (PBS injection). **: *p* < 0.01. (E) *Wwp1* expression in myocyte cells. Bubble size indicates the percentage of *Wwp1*-positive cells; color indicates average expression level.

In these three genes, Wwp1, an E3 ubiquitin ligase, is closely connected to the GO enrichment analysis. Based on the protein expression database (The Human Protein Atlas), analysis of WWP1 expression across different tissues revealed that skeletal muscle exhibited the highest expression level (Fig 2A). Analysis of single-cell RNA sequencing data from The Human Protein Atlas database [33] demonstrated that *WWP1* is primarily expressed in fast muscle cells within skeletal muscle (Fig 2B). To further explore the correlation among *Wwp1*, miR-191, and skeletal muscle, a skeletal muscle injury model was established in this study through CTX injection. RT-qPCR results showed that both miR-191 and *Wwp1* are associated with the skeletal muscle injury and repair process. Specifically, miR-191 was significantly downregulated, whereas *Wwp1* was significantly upregulated at day 3 post-CTX injection (Fig 2C-D).

As an independent validation, we analyzed a published single-nucleus RNA-seq dataset (GSE272412) derived from mouse tibialis anterior muscle at 3 days after CTX injury. By extracting the myocyte subpopulation, we found that in both aged and young mice, the proportion of *Wwp1*-positive myocytes and the average expression level of *Wwp1* within myocytes were significantly increased at 3 days post-CTX injury (Fig 2E). This result corroborates our qPCR findings (Fig 2D), further supporting the upregulation of *Wwp1* during skeletal muscle injury and regeneration.

### Transfection with miR-191 mimics impairs skeletal muscle differentiation

**Fig 3:**
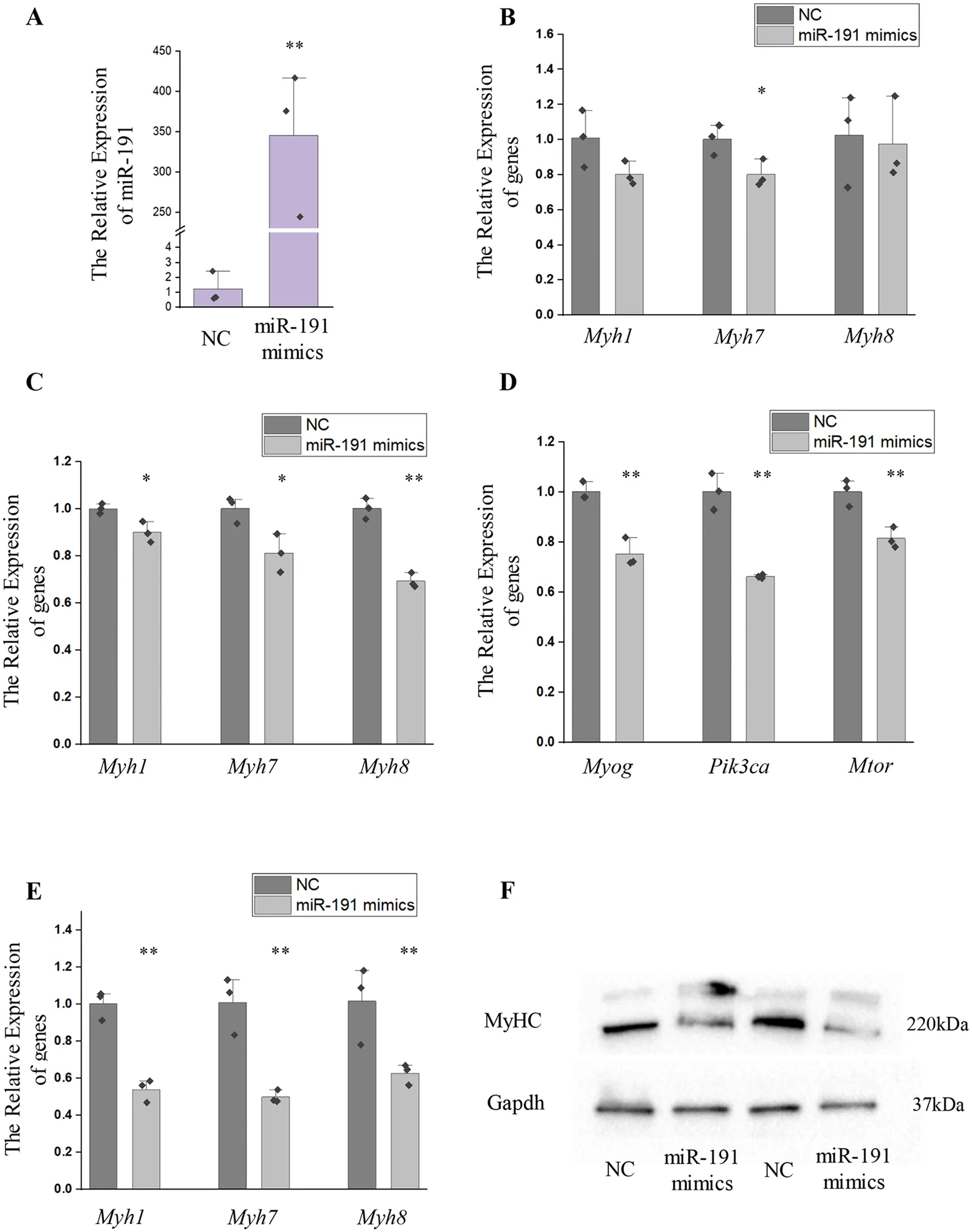
Transfection with miR-191 mimics affects myoblast differentiation. (A) RT-qPCR was used to verify the expression of miR-191 after transfection with miR-191 mimics (n = 3), **: *p* < 0.01. (B)-(C) miR-191 mimics were transfected before cell differentiation, and differentiation was induced when changing the culture medium after transfection. On day 3 (B) or day 5 (C) of differentiation, RT-qPCR was used to verify the expression of myosin heavy chain (*Myh1*, *Myh7*, *Myh8*) (n = 3), *: *p* < 0.05, **: *p* < 0.01. (D) miR-191 mimics were transfected before cell differentiation, and differentiation was induced when changing the culture medium after transfection. On day 3 of differentiation, RT-qPCR was used to verify the expression of *Myog*, PI3K (*Pik3ca*), and mTOR (*Mtor*) (n = 3), **: *p* < 0.01. (E) miR-191 mimics were transfected on day 3 of cell differentiation. On day 5 of differentiation, RT-qPCR was used to verify the expression of myosin heavy chain (*Myh1*, *Myh7*, *Myh8*) (n = 3), **: *p* < 0.01. (F) miR-191 mimics were transfected on day 3 of cell differentiation. On day 5 of differentiation, Western blot was used to verify the expression of MyHC. n = 4, shown is a representative result.

To further elucidate the regulatory role of miR-191 in skeletal muscle, we transfected C2C12 myoblasts with miR-191 mimics (Fig 3A), followed by induction of myogenic differentiation. We detected the expression changes of myosin heavy chain genes *Myh1*, *Myh7*, and *Myh8* at the mRNA level at 3 and 5 days post-differentiation. At day 3, *Myh7* was significantly downregulated, and at day 5, all three genes (*Myh1*, *Myh7*, and *Myh8*) exhibited significant downregulation compared to controls (Fig 3B-C).

So how does miR-191 influence the process of skeletal muscle differentiation? We investigated the myogenic regulatory factor Myog and critical components of the AKT signaling pathway. Results showed that miR-191 might influence differentiation by inhibiting *Myog*, *Pik3ca* (PI3K), and *Mtor* (mTOR) expression (Fig 3D). To further determine whether miR-191 affects the PI3K/mTOR pathway at the protein level, we detected the expression of p-AKT and total mTOR. Western blot analysis revealed that after transfection with miR-191 mimics, total mTOR protein levels were significantly decreased, while p-AKT (Ser473) levels showed no significant change (Fig S2 in S1 File). These findings suggest that miR-191 may be involved in the regulation of myogenic differentiation through downregulation of mTOR protein. During skeletal muscle differentiation, inhibiting mTOR may not only impair differentiation but also induce muscle atrophy [34,35]. To comprehensively characterize the impact of miR-191 on differentiation, we conducted miR-191 transfection on day 3 of differentiation and evaluated myosin heavy chain expression 48 h post-transfection (day 5 of differentiation). The detection results at both the mRNA and protein levels demonstrated that myosin expression exhibited a more pronounced suppression (Fig 3E-F).

**Fig 4:**
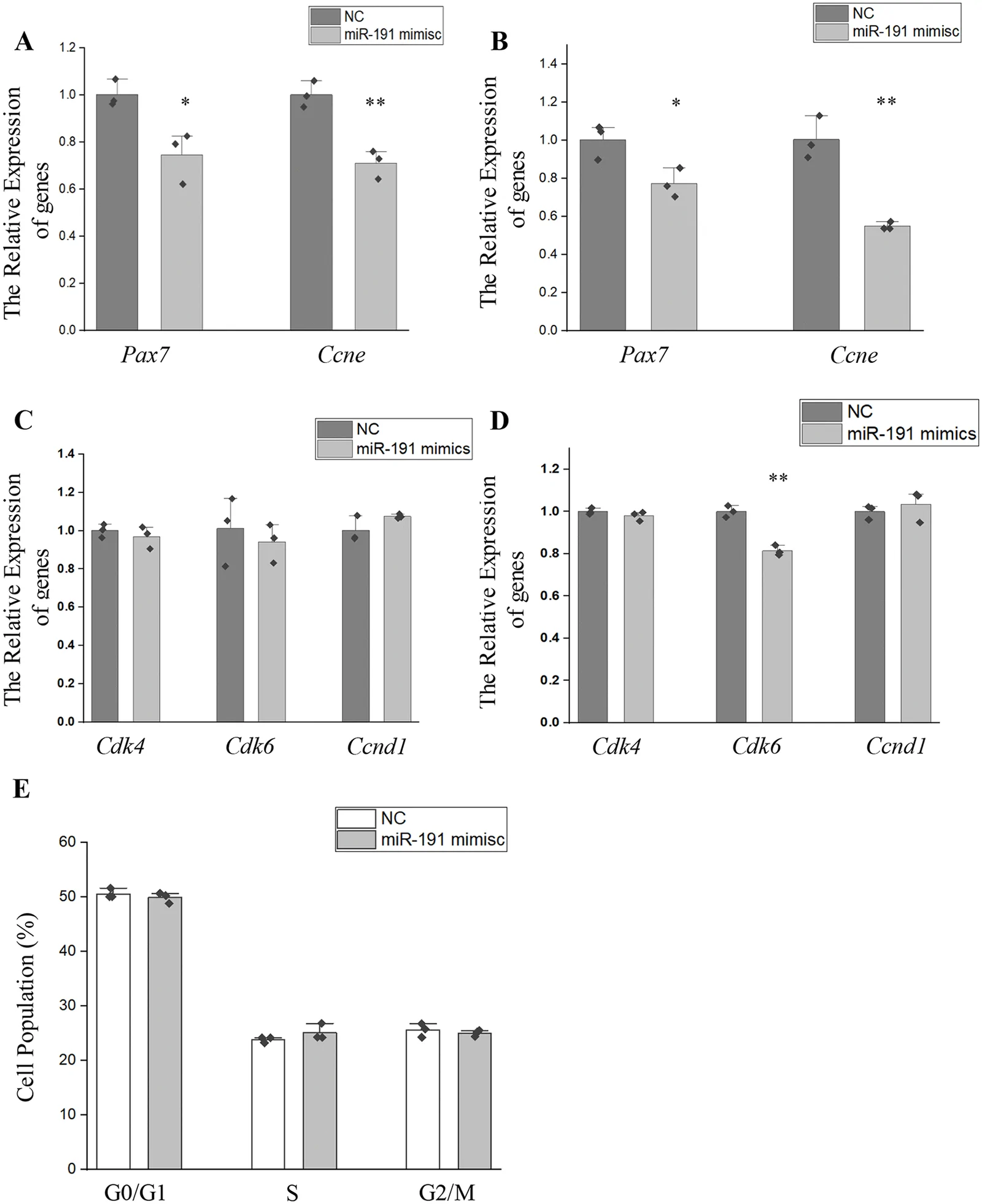
The effect of miR-191 on myoblast proliferation. (A)-(B) The expression of *Pax7* and *Ccne1* in myoblasts was detected by RT-qPCR 24 h or 36 h after transfection with miR-191 mimics (n = 3), *: *p* < 0.05, **: *p* < 0.01. (C)-(D) The expression of *Cdk4, Cdk6, and Ccnd1* in myoblasts was detected by RT-qPCR 24 h (C) or 36 h (D) after transfection with miR-191 mimics (n = 3), **: *p* < 0.01. (E) Flow cytometry was used to analyze the percentage of cells in the G0/G1, S, and G2/M phases (n = 3).

Regulation of myoblast proliferation impacts myoblast differentiation. To investigate whether miR-191 regulates differentiation by influencing proliferation, we examined the effects of miR-191 on myoblast proliferation. First, we transfected miR-191 mimics during the growth phase and analyzed the expression of the myogenic stem cell marker *Pax7* gene and cell cycle-related genes (*Ccne1*, *Cdk4*, *Cdk6*, *Ccnd1*) using RT-qPCR. Results demonstrated that while miR-191 exhibited an inhibitory effect on *Pax7*, its impact on cell cycle regulation was limited, showing only consistent suppression of *Ccne1* gene (Fig 4A-D). To further clarify the role of miR-191 in myoblast proliferation, we performed PI staining and cell cycle analysis through flow cytometry post-transfection. The findings revealed no significant effect of miR-191 on the cell cycle (Fig 4E).

**Fig 5:**
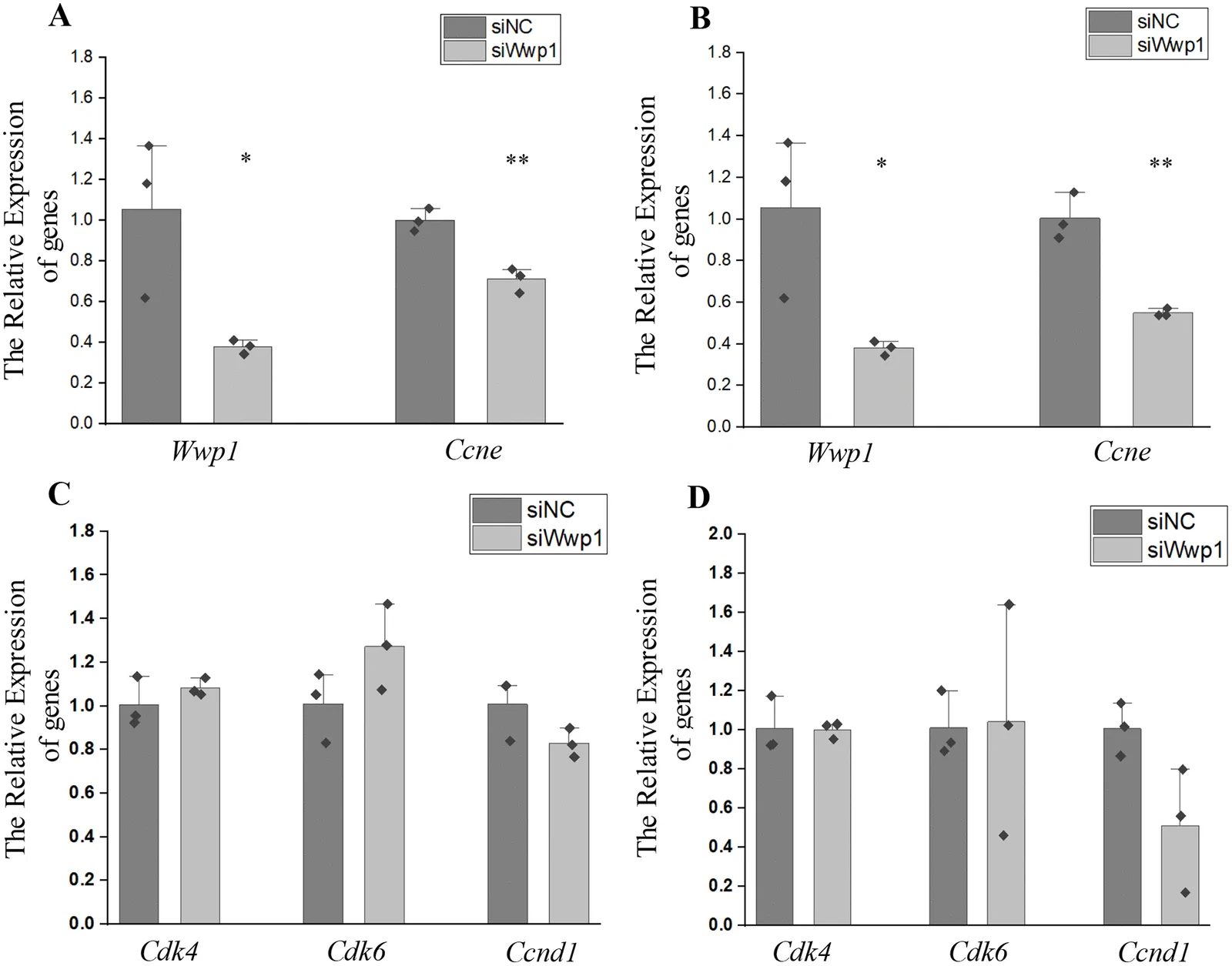
The effect of Wwp1 on myoblast proliferation. (A)-(B) The expression of *Pax7* and *Ccne1* in myoblasts was detected by RT-qPCR 24 h (A) or 36 h (B) after transfection with *Wwp1* siRNA (n = 3), *: *p* < 0.05, **: *p* < 0.01. (C)-(D) The expression of *Cdk4, Cdk6, and Ccnd1* in myoblasts was detected by RT-qPCR 24 h (C) or 36 h (D) after transfection with *Wwp1* siRNA (n = 3).

We further investigated the effect of Wwp1 on cellular proliferation regulation. We successfully suppressed Wwp1 expression by siRNA transfection (Fig 5A-B). Subsequent analysis of cell cycle-associated genes following *Wwp1* suppression revealed that only *Ccne1* expression was significantly inhibited (Fig 5A-D).

### *Wwp1* is a target gene of miR-191

**Fig 6:**
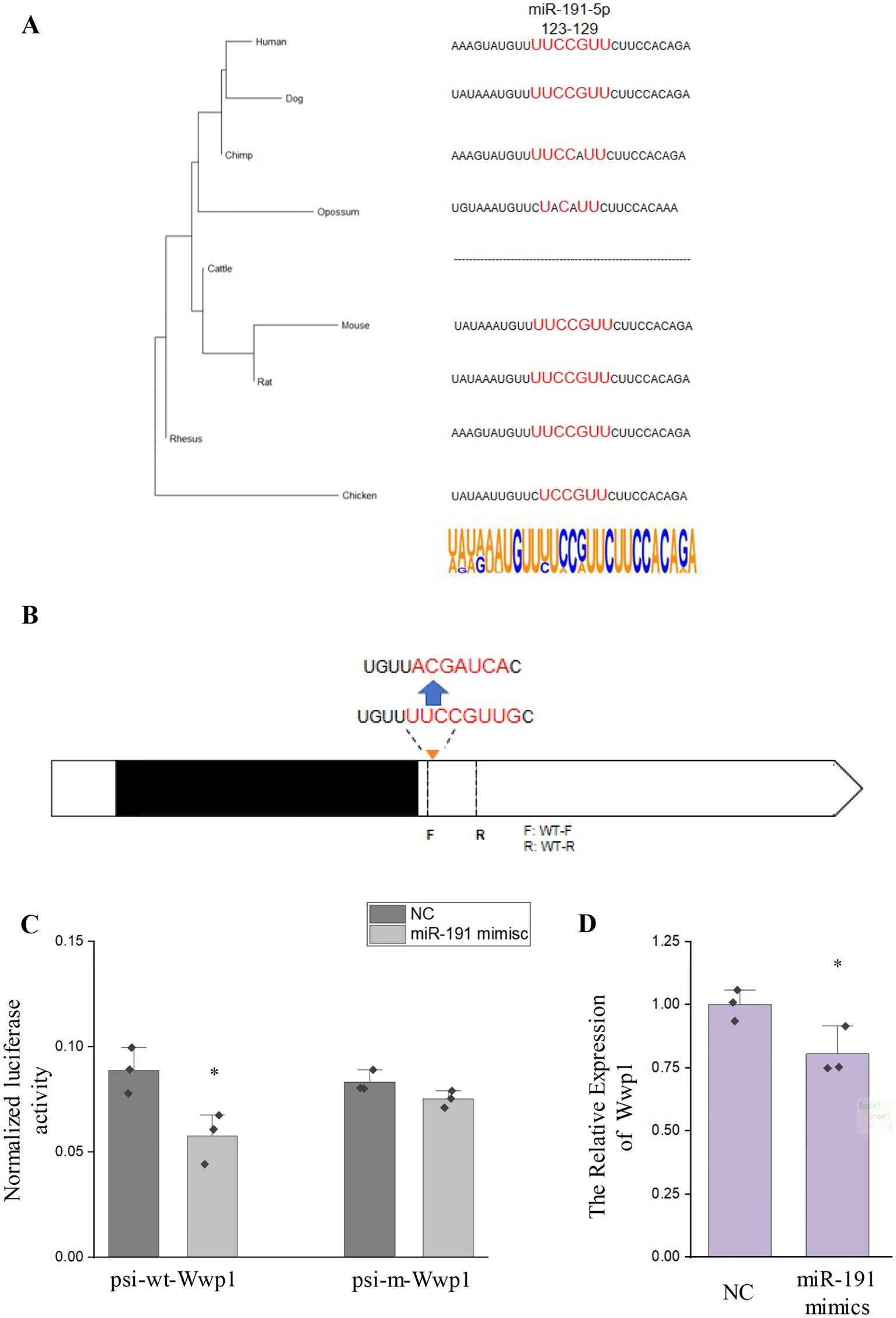
miR-191 directly regulates the *Wwp1* gene. (A) Analysis of 3’-UTRs of *Wwp1* gene and miR-191 binding sites across multiple species. (B) Schematic diagram of psi-wt-Wwp1 (wild-type) and psi-m-Wwp1 (mutant) vector construction. (C) Detection and relative activity analysis of Firefly luciferase and Renilla luciferase 24 h after co-transfection with miR-191 mimics and psiCHECK-2 vector (n = 3), *: *p* < 0.05. (D) The expression of *Wwp1* in myoblasts was detected by RT-qPCR 12 h after transfection with miR-191 mimics (n = 3), *: *p* < 0.05.

Finally, we explored whether miR-191 directly represses Wwp1 expression. To clarify the critical role of the 3’-untranslated region (3’-UTR) of the *Wwp1* gene in regulating its expression, we constructed a phylogenetic tree based on 3’-UTR sequences of *Wwp1* gene across multiple species (Fig 6A). The clustering in this phylogenetic tree showed discordance with traditional morphological classifications, suggesting that the 3’-UTR of *Wwp1* may have undergone unique selective pressures during evolution. Furthermore, analysis of miR-191 binding sites within the 3’-UTR of *Wwp1* revealed low sequence conservation across species (Fig 6A). Specifically, miR-191 binding sites were absent in cattle, opossum, and chicken, while potential binding sites for miR-191 were identified in the 3’-UTR of *Wwp1* in mice, rats, and humans.

**Fig 7:**
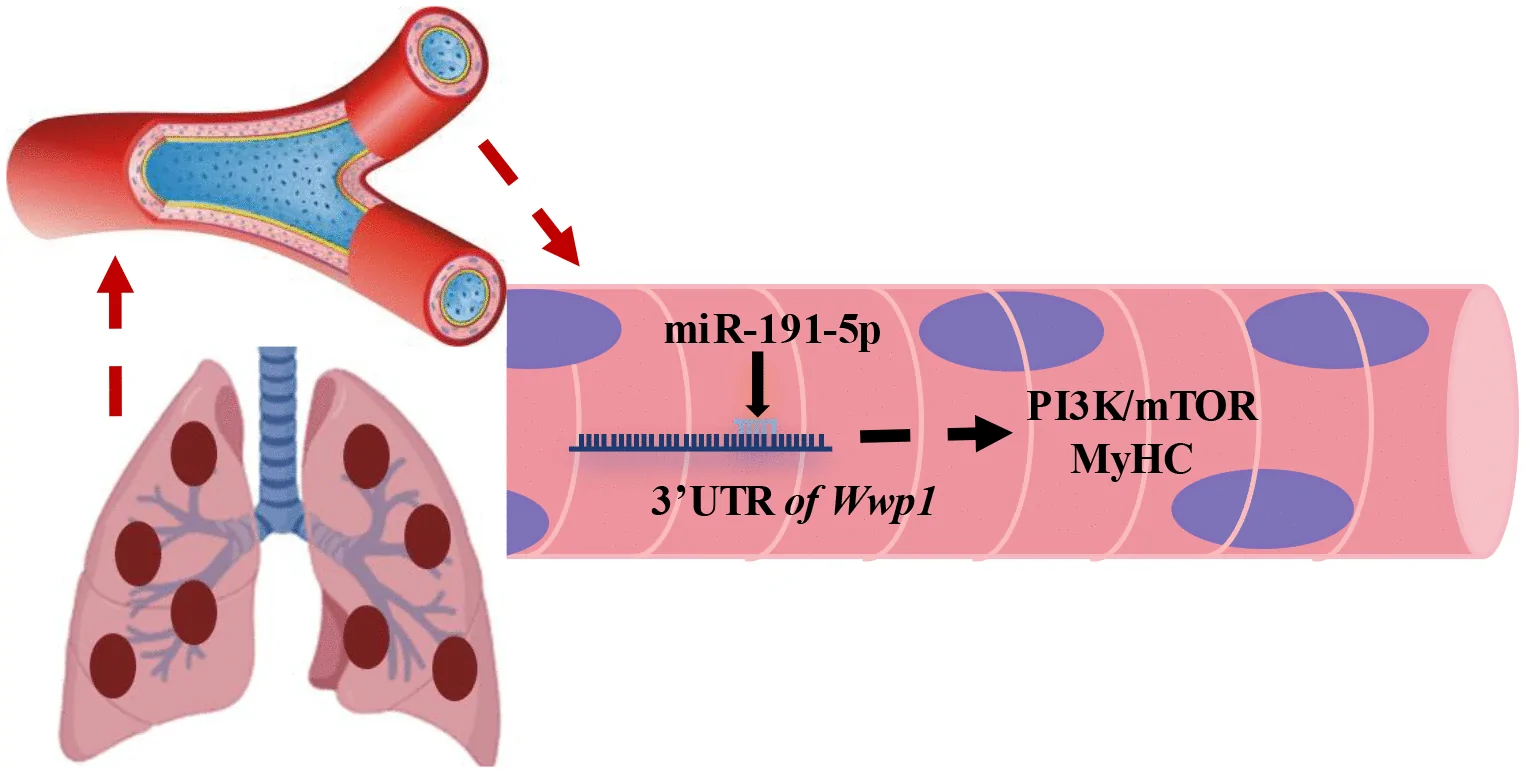
Graphical abstract.

To determine whether miR-191 can directly regulate the *Wwp1* gene, we cloned the 3’-untranslated region (3’-UTR) of *Wwp1* containing the miR-191 binding site into the multiple cloning site of the Renilla luciferase reporter gene in the psiCHECK-2 vector, constructing the psi-wt-Wwp1 plasmid. The mutation of the miR-191 binding site was achieved through overlap extension PCR. The mutated sequences were inserted into the psiCHECK-2 vector, resulting in the psi-m-Wwp1 plasmid (Fig. 6B). Subsequently, we co-transfected HEK293T cells with miR-191 mimics (or negative control, NC) and the constructed psiCHECK-2 vectors. After 24 h of transfection, we measured the activities of Firefly luciferase and Renilla luciferase (Fig 6C). By comparing the activities of the two luciferases, it was found that when miR-191 mimics were co-transfected with psi-wt-Wwp1, miR-191 suppressed the relative activity of Renilla luciferase. However, when co-transfected with psi-m-Wwp1, miR-191 had no such effect. This indicates that miR-191 directly targets the binding site in the 3’-UTR of *Wwp1*. To further elucidate the regulatory role of miR-191 on *Wwp1*, we examined the expression of the *Wwp1* gene at the mRNA level after transfection with miR-191 mimics. RT-qPCR results showed that miR-191 inhibited the expression of the *Wwp1* gene at the mRNA level (Fig 6D). miR-191 may regulate skeletal muscle differentiation by targeting the *Wwp1* gene (Fig 7).

## Discussion

Transfection with miR-191 mimics inhibited myoblast differentiation and regulated the mTOR pathway. Notably, transfection of miR-191 during C2C12 myoblast differentiation exerts a more pronounced inhibitory effect on differentiation markers Myh1/7/8 compared to transfection during the proliferative phase. The mTOR pathway demonstrates dual regulatory roles in skeletal muscle differentiation and metabolism [34,35]. This suggests that during the differentiation process, miR-191 may influence both skeletal muscle differentiation and metabolism by regulating the mTOR pathway.

The effect of miR-191 on myoblast proliferation was very limited. Although miR-191 inhibited *Pax7* gene expression, it showed no significant influence on multiple cell cycle-related genes, only stably suppressed the expression of *Ccne1*. Similar results were obtained when the expression of *Wwp1* was inhibited by siRNA. Previous studies have suggested that proliferation inhibition may promote differentiation [36], while others indicate that suppressed proliferation might not necessarily impact differentiation [22]. Importantly, miR-191 exerted regulatory effects on differentiation processes without affecting myoblast proliferation.

Wwp1 is an E3 ubiquitin ligase, but it differs from classic E3 ubiquitin ligases in skeletal muscle, such as Atrogin-1 and MuRF-1, which mediate the ubiquitination and degradation of myofibrillar proteins like MyHC, leading to muscle atrophy [7]. In contrast, Wwp1 inhibits atrophy by regulating Klf15 degradation [37]. Our analysis also showed that human WWP1 is differentially expressed in fast-twitch and slow-twitch muscles, suggesting it may be involved in fiber-type differences. Exploring the functions of Wwp1 can provide new insights into the molecular mechanisms underlying the distinct behaviors of fast and slow muscles during atrophy.

The regulation of miR-191 expression levels will be the focus of future research. miRNA expression levels are influenced by multifaceted regulatory mechanisms, including transcriptional activation, epigenetic regulation, miRNA editing, and sponge effects mediated by other non-coding RNAs [38]. Exploring these regulatory processes is very complex. For instance, Dicer1 participates in miRNA biogenesis, functional activation, and editing, yet different splice isoforms of Dicer1 can finely regulate specific miRNA populations [39]. Therefore, elucidating the regulatory mechanisms of miR-191 is intricate. Nonetheless, elucidating these regulatory mechanisms is indispensable for developing miRNA-based interventions and clarifying the molecular mechanisms of this biological process.

In summary, our findings suggest that miR-191 may modulate skeletal muscle differentiation by targeting Wwp1 and regulating the mTOR pathway. These findings may offer a perspective for understanding skeletal muscle atrophy associated with conditions such as COPD, and may also contribute to the understanding of the regulatory role of non-coding RNAs in skeletal muscle wasting.

## Supporting Information

S1 File This compressed file contains Supplementary Table S1 (primers for RT-qPCR), Supplementary Figures S1–S2 (effects of miR-191-5p expression in tumors/kidney and western blot analysis of mTOR/p-AKT), and the original uncropped western blot membranes. (ZIP)
