# Supplementary material for: miR-191 affects skeletal muscle differentiation by regulating Wwp1 in mouse myoblasts
Source: PLoS One. 2026 Sep 9;21(9):e0355370. doi: 10.1371/journal.pone.0355370 (PMC13557400; doi:10.1371/journal.pone.0355370)
Supplement: Supplementary file 1 [file pone.0355370.s003.docx]

Dear Editor.

We are very grateful to the editor and reviewers for their comments. We have carefully studied these valuable comments and answered all the questions. In the process, we are pleased to see that the quality of the article has been further improved and we believe that we can now meet the requirements of the journal. The point-to-point responses to the comments are listed as follows:

Responds to the editor’s comments:

Some conclusions are not really supported by the data.

**Response:** Thank you for your comment. We have carefully re-examined all conclusions drawn in the manuscript and revised the text to ensure that each conclusion is appropriately supported by the data.

For example:

“This study provides novel insights into the molecular mechanisms underlying skeletal muscle atrophy and establishes a theoretical foundation for understanding muscle wasting associated with conditions such as COPD.” was revised to “This study provides a theoretical basis for understanding muscle wasting associated with conditions such as COPD.”

“The GO enrichment results demonstrated that miR-191 is closely associated with ubiquitination processes,” was revised to “The GO enrichment results suggested that miR-191 may be involved in ubiquitination processes,”

All changes are highlighted in the revised manuscript. We believe the revised conclusions are now rigorous and fully supported by the data. Thank you for helping us improve the quality of our work.

1. Please ensure that your manuscript meets PLOS ONE's style requirements, including those for file naming.

**Response:** We have revised the formatting of the manuscript according to the journal’s style requirements.

2. To comply with PLOS One submissions requirements, in your Methods section, please provide additional information regarding the experiments involving animals and ensure you have included details on (1) methods of sacrifice, (2) methods of anesthesia and/or analgesia, and (3) efforts to alleviate suffering.

**Response:** Thank you for your reminder. We have now supplemented the “Injection of cardiotoxin (CTX)” subsection of the Materials and Methods with the following information:

Method of sacrifice: All mice were euthanized by cervical dislocation at the experimental endpoints (day 1 or day 3 post‑CTX injection/post‑PBS injection).

Method of anesthesia: Prior to CTX injection, mice were anesthetized by intraperitoneal administration of pentobarbital sodium (50 mg/kg).

Efforts to alleviate suffering: The CTX‑induced muscle injury model causes transient local inflammation and mild lameness, but no persistent severe pain was observed. Therefore, analgesics were not used to avoid interference with post‑injury inflammatory and regenerative processes. All mice were monitored twice daily for signs of distress, including body weight, fur condition, activity level, and behavior. If any unexpected severe distress had occurred, the animal would have been immediately euthanized; this was not required in our study. The animal protocol was approved by the Institutional Animal Care and Use Committee of Inner Mongolia University of Science and Technology (approval No. NMGKJDX‑2022‑9‑22).

These additions have been incorporated into the revised Materials and Methods section.

As follows:

“Materials and Methods

Injection of cardiotoxin (CTX)

Ten-week-old C57BL/6 mice were utilized to establish a cardiotoxin-induced skeletal muscle injury model. Mice were housed in an individually ventilated cage (IVC) system, with the temperature maintained between 22-26℃ and relative humidity between 45%-65%. Mice were maintained under a 12-hour light/12-hour dark cycle with ad libitum access to chow and water during the experiment. For cardiotoxin injection, mice were anesthetized via intraperitoneal administration of pentobarbital sodium (50 mg/kg). The fur overlying the tibialis anterior (TA) muscle was shaved, and the injection site was disinfected with an alcohol swab. Using a microsyringe, 100 μL of CTX solution (10 μM in PBS) was slowly injected into the tibialis anterior muscle. After injection, the mice were placed on a heating table until recovery from anesthesia and subsequently returned to their cages. Mice were randomly assigned to three groups: PBS‑injected control (n=2, harvested on day 1; n=3, harvested on day 3), CTX‑injected day 1 group (n=3, harvested on day 1), and CTX‑injected day 3 group (n=3, harvested on day 3). At the indicated endpoints, mice were euthanized by cervical dislocation. No analgesics were used to avoid interference with muscle regeneration; mice were monitored twice daily and no severe pain was observed. The animal protocol was approved by the Institutional Animal Care and Use Committee of Inner Mongolia University of Science and Technology (approval No. NMGKJDX‑2022‑9‑22).

”

3. Please update your submission to use the PLOS LaTeX template. The template and more information on our requirements for LaTeX submissions can be found at http://journals.plos.org/plosone/s/latex.

**Response:** Thank you for your suggestion. We wish to respectfully clarify that our manuscript was prepared in Microsoft Word (.docx), not in LaTeX. As PLOS ONE accepts submissions in Word format (as stated in the journal's submission guidelines), we have kept the manuscript in Word. We have carefully followed all other formatting requirements (including line numbers, file naming, and supporting information captions as requested). Please let us know if any further adjustments are needed. We appreciate your understanding. During the submission process, we have also learned how to use LaTeX, and if the manuscript is accepted, we can convert it to LaTeX format for final submission.

4. Funding Statement

**Response:** We have removed all funding-related text from the manuscript as requested. In the cover letter, we have stated that we wish to keep the existing Funding Statement unchanged.

5. Funding Statement

5.1 Review your statements relating to the author contributions,

5.2 Confirm in your cover letter that you agree with the following statement

**Response:** We have carefully reviewed and revised the author contributions section to ensure that the specific roles of each author are clearly and accurately indicated. These amendments have been made in the online submission form.

In addition, we confirm in this cover letter that we agree with the following statement:

“The funder provided support in the form of salaries for authors W.P., S.G., W.C., C.L., but did not have any additional role in the study design, data collection and analysis, decision to publish, or preparation of the manuscript. The specific roles of these authors are articulated in the ‘author contributions’ section”

6. Funding Statement

**Response:** Regarding the role of the funders, we confirm that the funders had no role in study design, data collection and analysis, decision to publish, or preparation of the manuscript. The funders provided financial support in the form of research grants only. we have included the Role of Funder statement in the cover letter.

7. Ethics statement should only appear in the Methods section of your manuscript.

**Response:** We have ensured that the ethics statement appears only in the Materials and Methods section of our manuscript. Specifically, it is included at the end of the “Injection of cardiotoxin (CTX)” subsection. The statement has been removed from any other section.

8. PLOS ONE now requires that authors provide the original uncropped and unadjusted images underlying all blot or gel results reported in a submission’s figures or Supporting Information files.

**Response:** We have complied with PLOS ONE’s blot/gel reporting requirements. The original blots were cut prior to antibody hybridization to optimize incubation conditions for multiple targets. The original and unadjusted images for Figure 3F have been provided in the Supporting Information. This has also been stated in the cover letter.

9. Please include captions for your Supporting Information files at the end of your manuscript.

**Response:** We have added captions for all Supporting Information files at the end of the manuscript (after the References section), in accordance with the journal’s guidelines. The captions are as follows:

“Supplementary Table. Primers used for quantitative reverse transcription PCR (RT-qPCR).

Supplementary Figure 1 Tumors and renal injuries influence the expression of miR-191-5p in serum or urine.”

10. If the reviewer comments include a recommendation to cite specific previously published works, please review and evaluate these publications to determine whether they are relevant and should be cited. There is no requirement to cite these works unless the editor has indicated otherwise.

**Response:** Not applicable. No citation recommendations were made by the reviewers.

Responds to the reviewer’s comments:

Reviewer #1:

1, the manuscript is not well written. It is full of grammar errors and should be thoroughly checked and corrected by language editor. Even the title is wrongly worded and should cover the fact that all the experiments were conducted in mice.

**Response:** Thank you for your reminder. We have thoroughly revised the language of the manuscript and hope that the revised version now meets the journal’s standards. We have also revised the title to reflect that all experiments were conducted in mice.

For example:

“The Mechanism of miR-191 Affecting Skeletal Muscle Differentiation by Regulating Wwp1.” was revised to “miR-191 Affects Skeletal Muscle Differentiation by Regulating Wwp1 in Mouse Myoblasts.”

“As crucial cargoes of extracellular vesicles, the regulatory roles of miRNAs in skeletal muscle atrophy associated with COPD, cachexia, and renal injury require further investigation.” was revised to “As crucial cargoes of extracellular vesicles, miRNAs play regulatory roles in skeletal muscle atrophy associated with COPD, cachexia, and renal injury require further investigation.”

2 Second, the materials and methods lack important details but contain irrelevant text that looks like copy/paste from reagent manuals.

**Response:** Thank you for your careful work. We have added the missing details (transfection medium, reagent, vector, animal numbers, anesthesia/euthanasia, luciferase assay steps, sample sources) and the Materials and Methods has been thoroughly revised.

For example:

“After injection, the mice were placed on a heating table until recovery from anesthesia and subsequently returned to their cages.” was revised to “After injection, the mice were placed on a heating table until recovery from anesthesia and subsequently returned to their cages. Mice were randomly assigned to three groups: PBS‑injected control (n=5, harvested on day 3), CTX‑injected day 1 group (n=3, harvested on day 1), and CTX‑injected day 3 group (n=3, harvested on day 3). At the indicated endpoints, mice were euthanized by cervical dislocation. No analgesics were used to avoid interference with muscle regeneration; mice were monitored twice daily and no severe pain was observed. The animal protocol was approved by the Institutional Animal Care and Use Committee of Inner Mongolia University of Science and Technology (approval No. NMGKJDX‑2022‑9‑22).”

3 miRNA mimics and siRNA should be better characterized and include targeting sites, sequences and catalogue numbers.
Provide the name of the “transfection reagent” used in the study
Provide the name of the “vector” used in the “Transfection protocol for HEK293T Cell”

**Response:** Thank you for your suggestion.

① We have added detailed information about the miRNA mimics and siRNA in the "Transfection" subsection of the Materials and Methods:

“The sequence of miR-191 mimics is CAACGGAAUCCCAAAAGCAGCUG. The catalog number of NC is miR1N0000002-1-5. The catalog number of siWwp1 is siB12329162409-1-5. The catalog number of siNC is siN0000001-1-5. All of the above nucleic acids were synthesized by RiboBio Co., Ltd. (Guangzhou, China).”

② We have added the name and catalog number of the transfection reagent in the "Transfection" subsection of the Materials and Methods:

“Lipo6000™, C0526.”

③ In the “Transfection protocol for HEK293T Cell” subsection of the Materials and Methods, we have clarified that the vector used is the psiCHECK-2 vector (psi-wt-Wwp1 or psi-m-Wwp1).

The psiCHECK™-2 vector (C8021, Promega) contains dual luciferase reporters (Renilla luciferase and Firefly luciferase) and is used for miRNA target validation. We have described the construction of the vector in the “Construction of psiCHECK-2 Vector” subsection of the Materials and Methods. In this revision, we have added the catalog number of this vector.

4 What medium was used in transfection procedures?

In general, the methods section, specifically transfection part is full of grammar errors, and should be shorten and corrected.

**Response:** Thank you for your careful work. The medium used during transfection was Opti-MEM™ (51985034, Gibco). We have added this information in the “Transfection” subsection of the Materials and Methods. Meanwhile, we have thoroughly reviewed and revised the Materials and Methods.

For example:

“During this incubation period, remove the old medium from the cells and add 1 mL of fresh serum-free medium.” was revised to “During this incubation period, the old medium was removed from the cells and 1 mL of fresh serum-free medium was added.”

5 How the “the activities of Renilla and Firefly luciferases” was measured?

**Response:** Thank you for your careful work. We have added the detection process for the activities of Renilla and Firefly luciferases in the “Transfection protocol for HEK293T Cell” subsection, as follows:

“Materials and Methods

Transfection protocol for HEK293T Cell:

Cells were harvested and fully lysed using 1×Passive Lysis Buffer (PLB) from the Dual-Luciferase® kit. Firefly luciferase activity was measured by adding Luciferase Assay Reagent II (LARII), and the luminescence was immediately read using a luminometer. Subsequently, Stop & Glo® Reagent was added to the same well to quench the firefly signal and simultaneously initiate the Renilla luciferase reaction, and the second reading was taken. The ratio of Renilla to Firefly luciferase activity was calculated for each well to normalize for transfection efficiency and cell number. This revised description aligns with standard protocols for the Dual-Luciferase® Reporter Assay System. We believe these additions fully address the reviewer’s concern. Thank you for helping us improve the methodological rigor of our manuscript.”

6 Which “Cells or homogenized tissues” were used in the study?

**Response:** Thank you for your suggestion. We have revised the RT-qPCR method section to clearly indicate what the cell samples and tissue samples refer to, as follows:

The cell samples refer to the myoblast cell line C2C12 used in the experiments shown in Figures 3–6; the tissue samples refer to the tibialis anterior muscle from the CTX-injected mouse experiment shown in Figures 2C–D.

7 What is the number of animals used in the study?

**Response:** Thank you for your advice. We thank the reviewer for the question. The number of animals used in this study has been indicated in the Methods section and in the legend of Figure 2C-D, as follows:

For cardiotoxin injection, mice were anesthetized via intraperitoneal administration of pentobarbital sodium (50 mg/kg). The fur overlying the tibialis anterior (TA) muscle was shaved, and the injection site was disinfected with an alcohol swab. Using a microsyringe, 100 μL of CTX solution (10 μM in PBS) was slowly injected into the tibialis anterior muscle. After injection, the mice were placed on a heating table until recovery from anesthesia and subsequently returned to their cages. Mice were randomly assigned to three groups: PBS‑injected control (n=2, harvested on day 1; n=3, harvested on day 3), CTX‑injected day 1 group (n=3, harvested on day 1), and CTX‑injected day 3 group (n=3, harvested on day 3).

8 What had happened to the animals after CTX injection?

**Response:** Thank you for your attention to animal welfare details. Following unilateral CTX injection into the TA muscle, the injected hindlimb showed transient local inflammation with mild lameness and limited mobility, but the mice could still move and feed on their own. Lameness peaked on day 1, began to improve on day 3, and returned to near-normal gait by day 7. No systemic abnormalities were observed during this period. In our original Materials and Methods, we only described the injection and recovery procedures, but did not provide detailed post-operative observations. The supplementary information is as follows:

“At the indicated endpoints, mice were euthanized by cervical dislocation. No analgesics were used to avoid interference with muscle regeneration; mice were monitored twice daily and no severe pain was observed. The animal protocol was approved by the Institutional Animal Care and Use Committee of Inner Mongolia University of Science and Technology (approval No. NMGKJDX‑2022‑9‑22).

”

9 The Supplementary Table with qPCR primers is missing

**Response:** Thank you for your careful work. In this revised submission, we have uploaded the Supplementary Table.

10 For how long was the differentiation continued? When the cells were transfected? When the RNA was isolated?

**Response:** Thank you for your suggestion. We have made corresponding additions in the figure legends.

For example:

“

**Figure 3 Transfection with miR-191 mimics affects myoblast differentiation.** (A) RT-qPCR was used to verify the expression of miR-191 after transfection with miR-191 mimics (n=3), **: *p* < 0.01. (B)-(C) miR-191 mimics were transfected before cell differentiation, and differentiation was induced when changing the culture medium after transfection. On (B) day 3 or (C) day 5 of differentiation, RT-qPCR was used to verify the expression of myosin heavy chain (*Myh1*, *Myh7*, *Myh8*) (n=3), *: *p* < 0.05, **: *p* < 0.01. (D) miR-191 mimics were transfected before cell differentiation, and differentiation was induced when changing the culture medium after transfection. On day 3 of differentiation, RT-qPCR was used to verify the expression of *Myog*, PI3K (*Pik3ca*), and mTOR (*Mtor*) (n=3), **: *p* < 0.01. (E) miR-191 mimics were transfected on day 3 of cell differentiation. On day 5 of differentiation, RT-qPCR was used to verify the expression of myosin heavy chain (*Myh1*, *Myh7*, *Myh8*) (n=3), **: *p* < 0.01. (F) miR-191 mimics were transfected on day 3 of cell differentiation. On day 5 of differentiation, Western blot was used to verify the expression of MyHC. n=4, shown is a representative result.”

11 This sentence lacks citations: “….tumor bearing mice or cancer patients, as well as in urine samples collected after kidney injury…”

**Response:** Thank you for pointing this out. We have deleted the "Data Source" section from the Methods, integrated that information into the main text, and provided the corresponding citations.

12 This sentence lacks citations: “….. Single-cell RNA sequencing data analysis demonstrated that WWP1 is primarily expressed in fast muscle cells…”

**Response:** Thank you very much for your careful review. We have revised the corresponding text and included the necessary citations.

13 In my opinion the paragraph “Data source” should be moved to the results section and adequately integrated with the description of the results visualized in Figures 1 and 2. Now, it is hard to tell where the data come form – own experiments or reanalysis of other results.

**Response:** Thank you very much for your valuable suggestion. We fully agree that clearly distinguishing the sources of data is essential for readers to understand the study. Accordingly, we have removed the "Data Source" paragraph from the Methods section and integrated its content into the descriptions of Figures 1, 2, and Supplementary Figure 1. In the Results section, we have explicitly used phrases such as "re-analyzed publicly available data" and "analysis of single‑cell RNA sequencing data from The Human Protein Atlas database" to make the data sources clear at a glance.

14 The authors mention “key transcriptional factors” but analyse only one – Myog, what about the others?

**Response:** Thank you for your opinion. We agree that skeletal muscle development involves multiple key transcription factors (such as MyoD, Myf5, Mrf4, and Myog). In this study, we focused on detecting Myog. Myog is a landmark transcription factor of the terminal stage of myogenic differentiation, and its expression level directly reflects whether cells have entered terminal differentiation. In contrast, MyoD and Myf5 primarily function during myoblast proliferation and commitment. Given that the main finding of this study is that miR-191 significantly inhibits terminal differentiation markers (Myh1/7/8), we focused on Myog as a representative indicator of differentiation status.

To avoid any misunderstanding, we have revised the original text by changing “key transcriptional factors” to “the myogenic regulatory factor Myog”.

15 Number of experiment repetitions is missing throughout the text and figure legends

**Response:** Thank you for your careful correction. We agree that clearly reporting the number of experimental replicates is essential for assessing the reliability and reproducibility of the data. We have also supplemented and standardized all figure legends by clearly indicating the number of independent biological replicates (n value) for each experiment. Accordingly, we have made the following revisions:

In the Statistical Analysis section of the Materials and Methods, we have added a general statement: “All experiments were performed with at least three independent biological replicates unless otherwise specified. The exact number of biological replicates (n) for each experiment is indicated in the corresponding figure legends.”

For the Western blot result (Figure 3F), we have indicated the number of replicates and added a more detailed description of the image: “Shown is a representative result.”

16 “overexpressed” should be changed to “transfected with”

**Response:** Thank you for your careful comment. We have changed all verb uses of “overexpressed” to “transfected with” throughout the manuscript, and kept “overexpression” only as a noun phrase where the context clearly indicates transfection as the method.

For example:

“we overexpressed miR-191 in C2C12 myoblast cell line by transfecting miR-191 mimics” was revised to “we transfected C2C12 myoblasts with miR-191 mimics”

“Overexpression of miR-191 affected myoblast differentiation” was revised to “Transfection with miR-191 mimics affects myoblast differentiation”

“We successfully knocked down Wwp1 using siRNA transfection” was revised to “We successfully suppressed Wwp1 expression by siRNA transfection”

17 In order to assess proliferation, why the cells were not simply counted in i.e. counting chambers?

**Response:** Thank you for the comment. In this study, we assessed the effect of miR-191 on C2C12 myoblast proliferation using the following methods: measuring the mRNA expression levels of proliferation‑related marker genes and performing cell cycle analysis by flow cytometry. While cell counting could reflect changes in total cell number, the methods we chose provide information on cell cycle distribution and specific molecular regulators of proliferation, which were the aspects we initially aimed to investigate. Our results ultimately showed that miR-191 had a significant inhibitory effect on differentiation but only a limited effect on proliferation. Therefore, we did not further confirm proliferation by cell counting in the present study. In future studies focusing specifically on proliferation, we will combine cell counting, flow cytometry, and other approaches to provide a more detailed description of proliferation.
