# Supplementary material for: miR-191 affects skeletal muscle differentiation by regulating Wwp1 in mouse myoblasts
Source: PLoS One. 2026 Sep 9;21(9):e0355370. doi: 10.1371/journal.pone.0355370 (PMC13557400; doi:10.1371/journal.pone.0355370)
Supplement: Supplementary file 2 [file pone.0355370.s004.docx]

Dear Editor.

Thank you for your continued guidance and for granting us additional time to revise our manuscript. We have now performed the requested experiments and analyses. Below we address each point point-by-point.

Responds to the editor’s comments:

Comment 1: Please verify altered mTOR activity at the protein level.

**Response:** Thank you for your suggestion. We have performed Western blot to examine the effect of miR-191 mimics on p-AKT (Ser473) and total mTOR protein levels in C2C12 myoblasts. As shown in the new Figure S2, transfection with miR-191 mimics significantly reduced total mTOR protein expression compared to the negative control. However, no significant change was detected in p-AKT (Ser473) levels. Densitometric analysis was performed using ImageJ. These data indicate that miR-191 downregulates mTOR protein, consistent with its inhibitory effect on Mtor mRNA (Fig 3D). Although p-AKT was not altered, the reduction in mTOR itself may contribute to the observed inhibition of myogenic differentiation. We have added this result to the Results section (lines 349–355) and Supplementary Figure 2.

As follows:

“To further determine whether miR-191 affects the PI3K/mTOR pathway at the protein level, we detected the expression of p-AKT and total mTOR. Western blot analysis revealed that after transfection with miR-191 mimics, total mTOR protein levels were significantly decreased, while p-AKT (Ser473) levels showed no significant change (Fig S2). These findings suggest that miR-191 may be involved in the regulation of myogenic differentiation through downregulation of mTOR protein.”

“

**Supplementary Figure 2** Western blot analysis of the effect of miR-191 mimics on mTOR and p-AKT protein expression. (A)-(B) miR-191 mimics were transfected before cell differentiation, and differentiation was induced when changing the culture medium after transfection. On day 3 of differentiation, Western blot was used to verify the expression of p-AKT (n=4, shown is a representative result ) and mTOR (n=3). Densitometric analysis was performed using ImageJ. ”

Comment 2: Please verify altered Wwp1 expression at the protein level.

**Response:** Thank you for your reminder. We fully agree that protein-level evidence would strengthen the conclusion. We tested several commercial anti-Wwp1 antibodies in both C2C12 lysates and mouse skeletal muscle tissues but were unable to obtain specific and reproducible bands under our experimental conditions. We acknowledge this technical limitation. As an alternative validation, we analyzed a published single-nucleus RNA-seq dataset (GSE272412) from mouse tibialis anterior muscle 3 days after CTX injury. By extracting the myocyte subpopulation, we found that in both aged and young mice, the proportion of Wwp1-positive myocytes and the average expression level of Wwp1 within myocytes were significantly increased at day 3 post‑injury (new Fig 2E). This result independently corroborates our qPCR findings (Fig 2D) and supports the upregulation of Wwp1 during skeletal muscle injury/regeneration. We have included this analysis in the Results section and described the approach in Materials and Methods.

As follows:

“Materials and Methods

Analysis of single-nucleus RNA-seq data from a mouse model of CTX-induced skeletal muscle injury (GSE272412)

We analyzed a publicly available single-nucleus RNA-seq dataset (GSE272412), in which mice were subjected to CTX-induced injury in the tibialis anterior muscle and sampled 3 days post-injury. From the expression matrix, we extracted the myocyte subpopulation. For each age group following CTX injury, we calculated (1) the proportion of Wwp1-positive myocytes and (2) the average expression level of Wwp1 within the myocyte population.”

“Results

As an independent validation, we analyzed a published single-nucleus RNA-seq dataset (GSE272412) derived from mouse tibialis anterior muscle at 3 days after CTX injury. By extracting the myocyte subpopulation, we found that in both aged and young mice, the proportion of Wwp1-positive myocytes and the average expression level of Wwp1 within myocytes were significantly increased at 3 days post-CTX injury (Fig. 2E). This result corroborates our qPCR findings (Fig. 2D), further supporting the upregulation of Wwp1 during skeletal muscle injury and regeneration.

(E) *Wwp1* expression in myocyte cells. Bubble size indicates the percentage of *Wwp1*-positive cells; color indicates average expression level.”

Sincerely,

Chuncheng Liu (on behalf of all authors)
