# Supplementary material for: miR-191 affects skeletal muscle differentiation by regulating Wwp1 in mouse myoblasts
Source: PLoS One. 2026 Sep 9;21(9):e0355370. doi: 10.1371/journal.pone.0355370 (PMC13557400; doi:10.1371/journal.pone.0355370)

Figure3

F

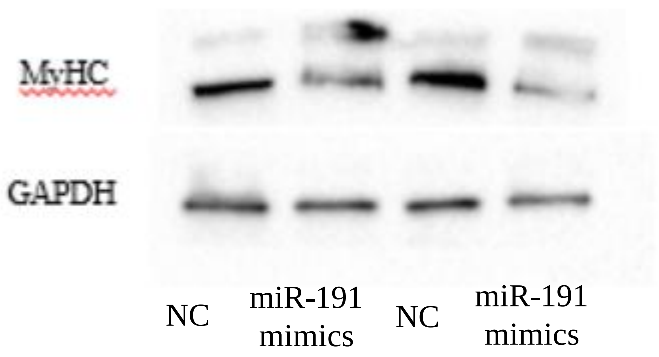
MyHC
GAPDH
NC
miR-191 mimics
NC
miR-191 mimics

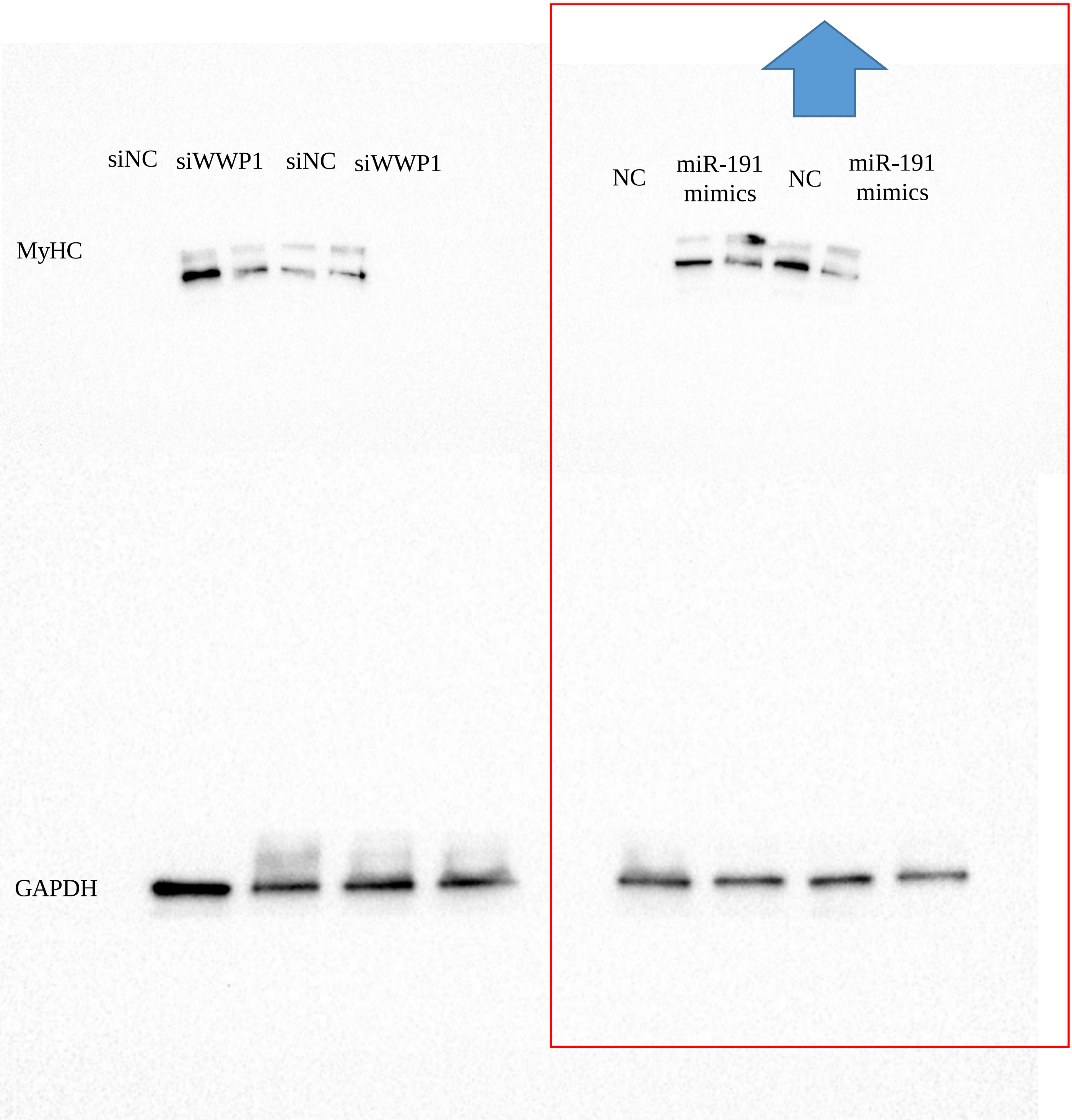
siNC
siWWP1
siNC
siWWP1
MyHC
GAPDH
NC
miR-191 mimics
NC
miR-191 mimics

Figure S2

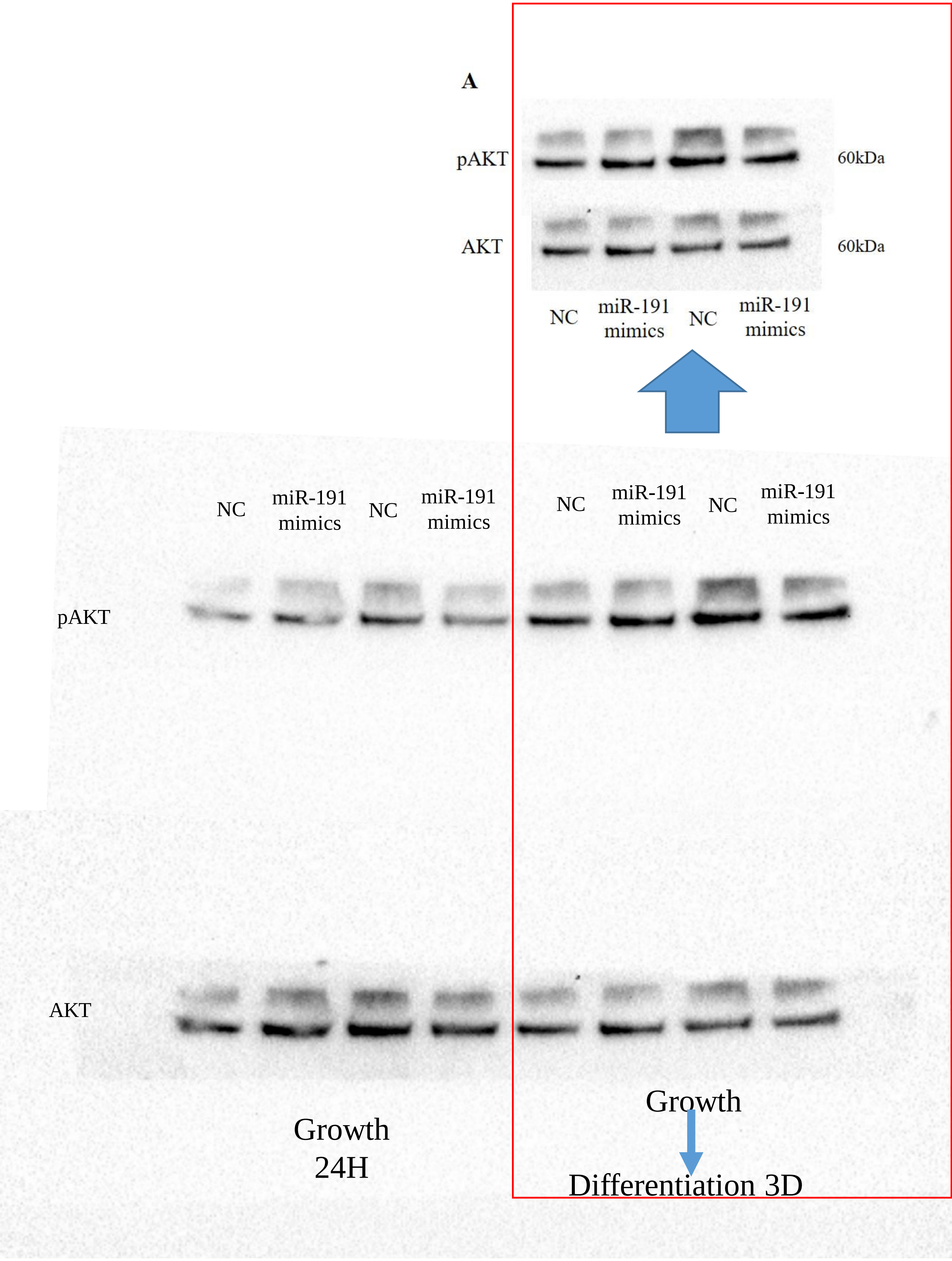
A
pAKT
60kDa
AKT
60kDa
NC
miR-191 mimics
NC
miR-191 mimics
NC
miR-191 mimics
NC
miR-191 mimics
NC
miR-191 mimics
NC
miR-191 mimics
pAKT
AKT
Growth 24H
Growth
Differentiation 3D

Figure S2

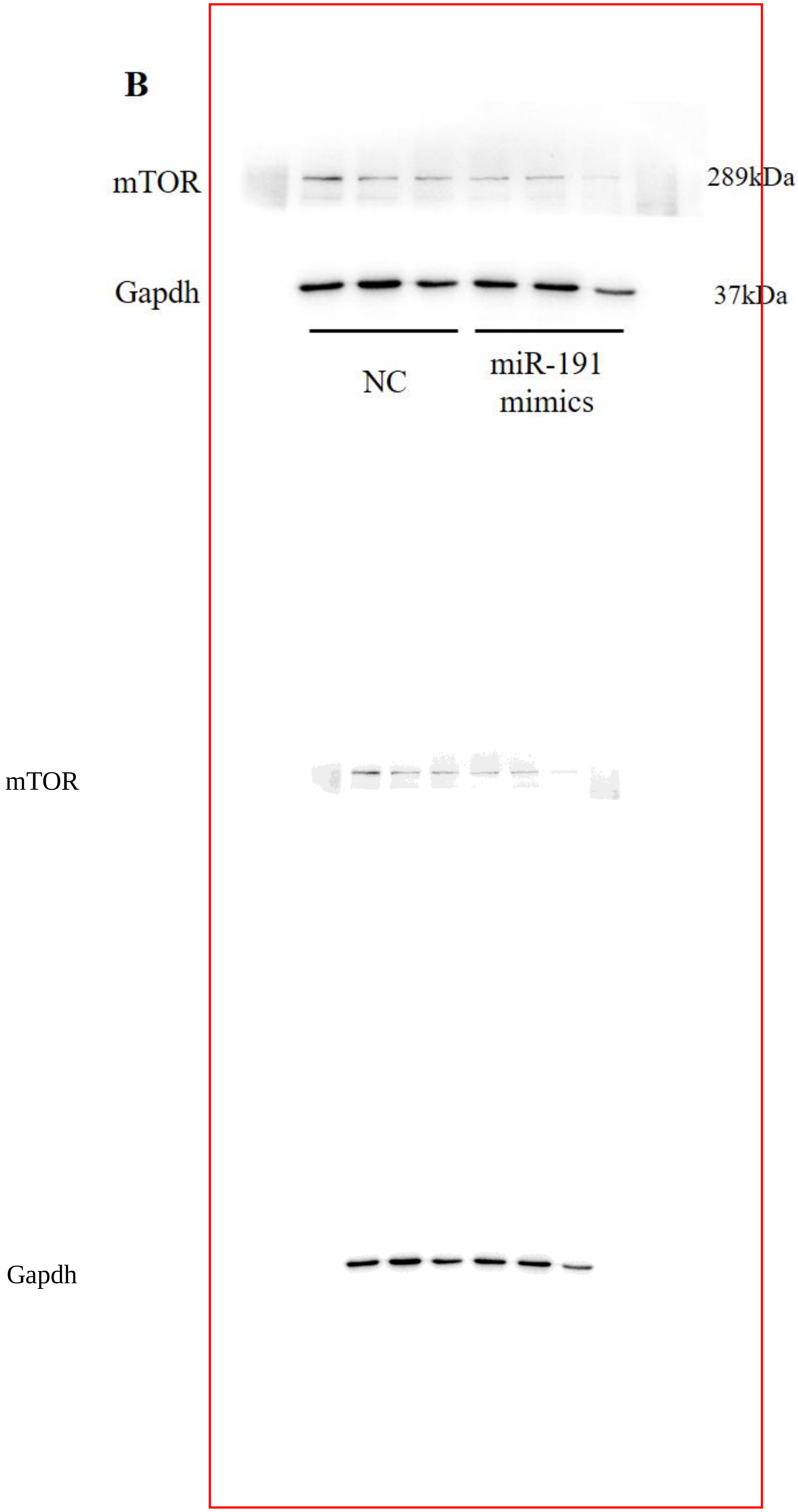

Supplement: Supplementary file 3 [file pone.0355370.s001.zip › Supporting Information/blot.pdf]
