# Supplementary material for: miR-191 affects skeletal muscle differentiation by regulating Wwp1 in mouse myoblasts
Source: PLoS One. 2026 Sep 9;21(9):e0355370. doi: 10.1371/journal.pone.0355370 (PMC13557400; doi:10.1371/journal.pone.0355370)
Supplement: Supplementary file 3 [file pone.0355370.s001.zip › Supporting Information/miR-191 Supplementary Figure1-2.docx]

**miR-191 Affects Skeletal Muscle Differentiation by Regulating Wwp1 in Mouse Myoblasts**

Wenhua Pu ^1,#^, Meimei Zhang ^2,#^, Siman Guo ^1^, Xiang Bao ^2^, Wenjia Cui ^1^, and Chuncheng Liu ^1,^*

^1^ Inner Mongolia Key Laboratory of Life Health and Bioinformatics, School of Life Science and Technology, Inner Mongolia University of Science & Technology, Baotou 014020, China

^2^ Department of Geriatrics, Ordos Central Hospital, Ordos, Inner Mongolia, China

^#^ Wenhua Pu and Meimei Zhang contributed equally to this work.

^*^ Correspondence: liuchuncheng.china@gmail.com (C.L.)

**Supplementary Fig 1. Tumors and renal injuries influence the expression of miR-191-5p in serum or urine.**

Tumor-bearing mice: Differentially expressed miRNAs in serum of tumor-bearing mice (MMTV-Her2/Neu). Non-small cell lung cancer patients: Differentially expressed miRNAs in plasma of non-small cell lung cancer patients. Ischemia-reperfusion injury: Differentially expressed miRNAs in urine of acute kidney injury rats induced by ischemia-reperfusion injury. Cisplatin-induced renal injury: Differentially expressed miRNAs in the urine of rats with cisplatin-induced proximal tubular injury.

**Supplementary Fig 2. Western blot analysis of the effect of miR-191 mimics on mTOR and p-AKT protein expression.**

(A)-(B) miR-191 mimics were transfected before cell differentiation, and differentiation was induced when changing the culture medium after transfection. On day 3 of differentiation, Western blot was used to verify the expression of p-AKT (n=4, shown is a representative result ) and mTOR (n=3). Densitometric analysis was performed using ImageJ.
