# Supplementary material for: miR-191 affects skeletal muscle differentiation by regulating Wwp1 in mouse myoblasts
Source: PLoS One. 2026 Sep 9;21(9):e0355370. doi: 10.1371/journal.pone.0355370 (PMC13557400; doi:10.1371/journal.pone.0355370)
Supplement: Peer review history

# Peer review: Decision Letter 0

**DOI:** 10.1371/journal.pone.0355370.r001

**Authors:** Hongkyun Kim

30 Mar 2026

Dear Dr. Liu,

Thank you for submitting your manuscript to PLOS ONE. After careful consideration, we feel that it has merit but does not fully meet PLOS ONE’s publication criteria as it currently stands. Therefore, we invite you to submit a revised version of the manuscript that addresses the points raised during the review process.

I apologize that the review process took so long. As the reviewer indicated, I concur that your manuscript can be improved by professional editing. I feel that some conclusions are not really supported by the data. For example, Wwp1 expression is increased in 3 days after CTX injection. However, these data are from RT-qPCR; therefore, it is difficult to conclude whether Wwp1 is increased at the protein level. Moreover, you conclude that miR-191 regulates PI3K/mTOR pathway based on RT-qPCR data. Particularly, the difference in the expression level is a bare minimum with only three samples. Given that there are other options to measure mTOR activity, I recommend that you consider them.

Please submit your revised manuscript by May 14 2026 11:59PM. If you will need more time than this to complete your revisions, please reply to this message or contact the journal office at plosone@plos.org. When you're ready to submit your revision, log on to https://www.editorialmanager.com/pone/ and select the 'Submissions Needing Revision' folder to locate your manuscript file.

- A letter that responds to each point raised by the academic editor and reviewer(s). You should upload this letter as a separate file labeled 'Response to Reviewers'.
- A marked-up copy of your manuscript that highlights changes made to the original version. You should upload this as a separate file labeled 'Revised Manuscript with Track Changes'.
- An unmarked version of your revised paper without tracked changes. You should upload this as a separate file labeled 'Manuscript'.

If you would like to make changes to your financial disclosure, please include your updated statement in your cover letter. Guidelines for resubmitting your figure files are available below the reviewer comments at the end of this letter.

If applicable, we recommend that you deposit your laboratory protocols in protocols.io to enhance the reproducibility of your results. Protocols.io assigns your protocol its own identifier (DOI) so that it can be cited independently in the future. For instructions see: https://journals.plos.org/plosone/s/submission-guidelines#loc-laboratory-protocols. Additionally, PLOS ONE offers an option for publishing peer-reviewed Lab Protocol articles, which describe protocols hosted on protocols.io. Read more information on sharing protocols at https://plos.org/protocols?utm_medium=editorial-email&utm_source=authorletters&utm_campaign=protocols.

As the corresponding author, your ORCID iD is verified in the submission system and will appear in the published article. PLOS supports the use of ORCID, and we encourage all coauthors to register for an ORCID iD and use it as well. Please encourage your coauthors to verify their ORCID iD within the submission system before final acceptance, as unverified ORCID iDs will not appear in the published article. *Only*  the individual author can complete the verification step; PLOS staff *cannot*  verify ORCID iDs on behalf of authors.

We look forward to receiving your revised manuscript.

Kind regards,

Hongkyun Kim

Academic Editor

PLOS One

Journal Requirements:

When submitting your revision, we need you to address these additional requirements.

1. Please ensure that your manuscript meets PLOS ONE's style requirements, including those for file naming. The PLOS ONE style templates can be found at

https://journals.plos.org/plosone/s/file?id=wjVg/PLOSOne_formatting_sample_main_body.pdf and

https://journals.plos.org/plosone/s/file?id=ba62/PLOSOne_formatting_sample_title_authors_affiliations.pdf

2. To comply with PLOS One submissions requirements, in your Methods section, please provide additional information regarding the experiments involving animals and ensure you have included details on (1) methods of sacrifice, (2) methods of anesthesia and/or analgesia, and (3) efforts to alleviate suffering.

3. Please update your submission to use the PLOS LaTeX template. The template and more information on our requirements for LaTeX submissions can be found at http://journals.plos.org/plosone/s/latex.

4. Thank you for stating the following in the Acknowledgments Section of your manuscript:

“This research was funded by the Fundamental Research Funds for Inner Mongolia University of Science & Technology, grant number 2023RCTD019 and 2023YXXS027; the Natural Science Foundation of Inner Mongolia, grant number 2025MS03036; the 2025 Inner Mongolia Key Laboratory of Life Health and Bioinformatics Project, grant number 2025KYPT0135.”

We note that you have provided funding information that is not currently declared in your Funding Statement. However, funding information should not appear in the Acknowledgments section or other areas of your manuscript. We will only publish funding information present in the Funding Statement section of the online submission form.

Please remove any funding-related text from the manuscript and let us know how you would like to update your Funding Statement. Currently, your Funding Statement reads as follows:

“This research was funded by the Fundamental Research Funds for Inner Mongolia University of Science & Technology, grant number 2023RCTD019 and 2023YXXS027; the Natural Science Foundation of Inner Mongolia, grant number 2025MS03036; the 2025 Inner Mongolia Key Laboratory of Life Health and Bioinformatics Project, grant number 2025KYPT0135.”

Please include your amended statements within your cover letter; we will change the online submission form on your behalf.

5. Thank you for stating the following financial disclosure:

“This research was funded by the Fundamental Research Funds for Inner Mongolia University of Science & Technology, grant number 2023RCTD019 and 2023YXXS027; the Natural Science Foundation of Inner Mongolia, grant number 2025MS03036; the 2025 Inner Mongolia Key Laboratory of Life Health and Bioinformatics Project, grant number 2025KYPT0135.”

We note that one or more of the authors is affiliated with the funding organization, indicating the funder may have had some role in the design, data collection, analysis or preparation of your manuscript for publication; in other words, the funder played an indirect role through the participation of the co-authors. If the funding organization did not play a role in the study design, data collection and analysis, decision to publish, or preparation of the manuscript and only provided financial support in the form of authors' salaries and/or research materials, please do the following:

1. Review your statements relating to the author contributions, and ensure you have specifically and accurately indicated the role(s) that these authors had in your study. These amendments should be made in the online form.

2. Confirm in your cover letter that you agree with the following statement, and we will change the online submission form on your behalf:

“The funder provided support in the form of salaries for authors [insert relevant initials], but did not have any additional role in the study design, data collection and analysis, decision to publish, or preparation of the manuscript. The specific roles of these authors are articulated in the ‘author contributions’ section.

6. Thank you for stating the following financial disclosure:

“This research was funded by the Fundamental Research Funds for Inner Mongolia University of Science & Technology, grant number 2023RCTD019 and 2023YXXS027; the Natural Science Foundation of Inner Mongolia, grant number 2025MS03036; the 2025 Inner Mongolia Key Laboratory of Life Health and Bioinformatics Project, grant number 2025KYPT0135.”

Please state what role the funders took in the study.  If the funders had no role, please state: "The funders had no role in study design, data collection and analysis, decision to publish, or preparation of the manuscript."

If this statement is not correct you must amend it as needed.

Please include this amended Role of Funder statement in your cover letter; we will change the online submission form on your behalf.

7. Your ethics statement should only appear in the Methods section of your manuscript. If your ethics statement is written in any section besides the Methods, please move it to the Methods section and delete it from any other section. Please ensure that your ethics statement is included in your manuscript, as the ethics statement entered into the online submission form will not be published alongside your manuscript.

8. PLOS ONE now requires that authors provide the original uncropped and unadjusted images underlying all blot or gel results reported in a submission’s figures or Supporting Information files. This policy and the journal’s other requirements for blot/gel reporting and figure preparation are described in detail at https://journals.plos.org/plosone/s/figures#loc-blot-and-gel-reporting-requirements and https://journals.plos.org/plosone/s/figures#loc-preparing-figures-from-image-files. When you submit your revised manuscript, please ensure that your figures adhere fully to these guidelines and provide the original underlying images for all blot or gel data reported in your submission. See the following link for instructions on providing the original image data: https://journals.plos.org/plosone/s/figures#loc-original-images-for-blots-and-gels.

In your cover letter, please note whether your blot/gel image data are in Supporting Information or posted at a public data repository, provide the repository URL if relevant, and provide specific details as to which raw blot/gel images, if any, are not available. Email us at plosone@plos.org if you have any questions.

9. Please include captions for your Supporting Information files at the end of your manuscript, and update any in-text citations to match accordingly. Please see our Supporting Information guidelines for more information: http://journals.plos.org/plosone/s/supporting-information.

10. If the reviewer comments include a recommendation to cite specific previously published works, please review and evaluate these publications to determine whether they are relevant and should be cited. There is no requirement to cite these works unless the editor has indicated otherwise.

[Note: HTML markup is below. Please do not edit.]

Reviewers' comments:

Reviewer's Responses to Questions

**Comments to the Author**

1. Is the manuscript technically sound, and do the data support the conclusions?

Reviewer #1: No

**********

2. Has the statistical analysis been performed appropriately and rigorously?

Reviewer #1: I Don't Know

**********

3. Have the authors made all data underlying the findings in their manuscript fully available?

The PLOS Data policy

Reviewer #1: No

**********

4. Is the manuscript presented in an intelligible fashion and written in standard English?

Reviewer #1: No

**********

Reviewer #1: The manuscript entitled “The Mechanism of miR-191 Affecting Skeletal Muscle Differentiation by Regulating Wwp1” is interesting, however, its suffers from some flaws and multiple issues should be addressed before publication. First of all, the manuscript is not well written. It is full of grammar errors and should be thoroughly checked and corrected by language editor. Even the title is wrongly worded and should cover the fact that all the experiments were conducted in mice.

Second, the materials and methods lack important details but contain irrelevant text that looks like copy/paste from reagent manuals.

Details:

miRNA mimics and siRNA should be better characterized and include targeting sites, sequences and catalogue numbers.

Provide the name of the “transfection reagent” used in the study

Provide the name of the “vector” used in the “Transfection protocol for HEK293T Cell”

What medium was used in transfection procedures?

In general, the methods section, specifically transfection part is full of grammar errors, and should be shorten and corrected.

How the “the activities of Renilla and Firefly luciferases” was measured?

Which “Cells or homogenized tissues” were used in the study?

What is the number of animals used in the study?

What had happened to the animals after CTX injection?

The Supplementary Table with qPCR primers is missing

For how long was the differentiation continued? When the cells were transfected? When the RNA was isolated?

This sentence lacks citations: “….tumor bearing mice or cancer patients, as well as in urine samples collected after kidney injury…”

This sentence lacks citations: “….. Single-cell RNA sequencing data analysis demonstrated that WWP1 is primarily expressed in fast muscle cells…”

In my opinion the paragraph “Data source” should be moved to the results section and adequately integrated with the description of the results visualized in Figures 1 and 2. Now, it is hard to tell where the data come form – own experiments or reanalysis of other results.

The authors mention “key transcriptional factors” but analyse only one – Myog, what about the others?

Number of experiment repetitions is missing throughout the text and figure legends

“overexpressed” should be changed to “transfected with”

In order to assess proliferation, why the cells were not simply counted in i.e. counting chambers?

**********

what does this mean?). If published, this will include your full peer review and any attached files.

If you choose “no”, your identity will remain anonymous but your review may still be made public.

**Do you want your identity to be public for this peer review?**  For information about this choice, including consent withdrawal, please see our Privacy Policy

Reviewer #1: No

**********

[NOTE: If reviewer comments were submitted as an attachment file, they will be attached to this email and accessible via the submission site. Please log into your account, locate the manuscript record, and check for the action link "View Attachments". If this link does not appear, there are no attachment files.]

To ensure your figures meet our technical requirements, please review our figure guidelines: https://journals.plos.org/plosone/s/figures

You may also use PLOS’s free figure tool, NAAS, to help you prepare publication quality figures: https://journals.plos.org/plosone/s/figures#loc-tools-for-figure-preparation.

NAAS will assess whether your figures meet our technical requirements by comparing each figure against our figure specifications.

# Author comment: Author response to Decision Letter 1

**DOI:** 10.1371/journal.pone.0355370.r002

17 Apr 2026

Responds to the editor’s comments:

Some conclusions are not really supported by the data.

Response: Thank you for your comment. We have carefully re-examined all conclusions drawn in the manuscript and revised the text to ensure that each conclusion is appropriately supported by the data.

For example:

“This study provides novel insights into the molecular mechanisms underlying skeletal muscle atrophy and establishes a theoretical foundation for understanding muscle wasting associated with conditions such as COPD.” was revised to “This study provides a theoretical basis for understanding muscle wasting associated with conditions such as COPD.”

“The GO enrichment results demonstrated that miR-191 is closely associated with ubiquitination processes,” was revised to “The GO enrichment results suggested that miR-191 may be involved in ubiquitination processes,”

All changes are highlighted in the revised manuscript. We believe the revised conclusions are now rigorous and fully supported by the data. Thank you for helping us improve the quality of our work.

1. Please ensure that your manuscript meets PLOS ONE's style requirements, including those for file naming.

Response: We have revised the formatting of the manuscript according to the journal’s style requirements.

2. To comply with PLOS One submissions requirements, in your Methods section, please provide additional information regarding the experiments involving animals and ensure you have included details on (1) methods of sacrifice, (2) methods of anesthesia and/or analgesia, and (3) efforts to alleviate suffering.

Response: Thank you for your reminder. We have now supplemented the “Injection of cardiotoxin (CTX)” subsection of the Materials and Methods with the following information:

Method of sacrifice: All mice were euthanized by cervical dislocation at the experimental endpoints (day 1 or day 3 post‑CTX injection/post‑PBS injection).

Method of anesthesia: Prior to CTX injection, mice were anesthetized by intraperitoneal administration of pentobarbital sodium (50 mg/kg).

Efforts to alleviate suffering: The CTX‑induced muscle injury model causes transient local inflammation and mild lameness, but no persistent severe pain was observed. Therefore, analgesics were not used to avoid interference with post‑injury inflammatory and regenerative processes. All mice were monitored twice daily for signs of distress, including body weight, fur condition, activity level, and behavior. If any unexpected severe distress had occurred, the animal would have been immediately euthanized; this was not required in our study. The animal protocol was approved by the Institutional Animal Care and Use Committee of Inner Mongolia University of Science and Technology (approval No. NMGKJDX‑2022‑9‑22).

These additions have been incorporated into the revised Materials and Methods section.

As follows:

“Materials and Methods

Injection of cardiotoxin (CTX)

Ten-week-old C57BL/6 mice were utilized to establish a cardiotoxin-induced skeletal muscle injury model. Mice were housed in an individually ventilated cage (IVC) system, with the temperature maintained between 22-26℃ and relative humidity between 45%-65%. Mice were maintained under a 12-hour light/12-hour dark cycle with ad libitum access to chow and water during the experiment. For cardiotoxin injection, mice were anesthetized via intraperitoneal administration of pentobarbital sodium (50 mg/kg). The fur overlying the tibialis anterior (TA) muscle was shaved, and the injection site was disinfected with an alcohol swab. Using a microsyringe, 100 μL of CTX solution (10 μM in PBS) was slowly injected into the tibialis anterior muscle. After injection, the mice were placed on a heating table until recovery from anesthesia and subsequently returned to their cages. Mice were randomly assigned to three groups: PBS‑injected control (n=2, harvested on day 1; n=3, harvested on day 3), CTX‑injected day 1 group (n=3, harvested on day 1), and CTX‑injected day 3 group (n=3, harvested on day 3). At the indicated endpoints, mice were euthanized by cervical dislocation. No analgesics were used to avoid interference with muscle regeneration; mice were monitored twice daily and no severe pain was observed. The animal protocol was approved by the Institutional Animal Care and Use Committee of Inner Mongolia University of Science and Technology (approval No. NMGKJDX‑2022‑9‑22).”

3. Please update your submission to use the PLOS LaTeX template. The template and more information on our requirements for LaTeX submissions can be found at http://journals.plos.org/plosone/s/latex.

Response: Thank you for your suggestion. We wish to respectfully clarify that our manuscript was prepared in Microsoft Word (.docx), not in LaTeX. As PLOS ONE accepts submissions in Word format (as stated in the journal's submission guidelines), we have kept the manuscript in Word. We have carefully followed all other formatting requirements (including line numbers, file naming, and supporting information captions as requested). Please let us know if any further adjustments are needed. We appreciate your understanding. During the submission process, we have also learned how to use LaTeX, and if the manuscript is accepted, we can convert it to LaTeX format for final submission.

4. Funding Statement

Response: We have removed all funding-related text from the manuscript as requested. In the cover letter, we have stated that we wish to keep the existing Funding Statement unchanged.

5. Funding Statement

5.1 Review your statements relating to the author contributions,

5.2 Confirm in your cover letter that you agree with the following statement

Response: We have carefully reviewed and revised the author contributions section to ensure that the specific roles of each author are clearly and accurately indicated. These amendments have been made in the online submission form.

In addition, we confirm in this cover letter that we agree with the following statement:

“The funder provided support in the form of salaries for authors W.P., S.G., W.C., C.L., but did not have any additional role in the study design, data collection and analysis, decision to publish, or preparation of the manuscript. The specific roles of these authors are articulated in the ‘author contributions’ section”

6. Funding Statement

Response: Regarding the role of the funders, we confirm that the funders had no role in study design, data collection and analysis, decision to publish, or preparation of the manuscript. The funders provided financial support in the form of research grants only. we have included the Role of Funder statement in the cover letter.

7. Ethics statement should only appear in the Methods section of your manuscript.

Response: We have ensured that the ethics statement appears only in the Materials and Methods section of our manuscript. Specifically, it is included at the end of the “Injection of cardiotoxin (CTX)” subsection. The statement has been removed from any other section.

8. PLOS ONE now requires that authors provide the original uncropped and unadjusted images underlying all blot or gel results reported in a submission’s figures or Supporting Information files.

Response: We have complied with PLOS ONE’s blot/gel reporting requirements. The original blots were cut prior to antibody hybridization to optimize incubation conditions for multiple targets. The original and unadjusted images for Figure 3F have been provided in the Supporting Information. This has also been stated in the cover letter.

9. Please include captions for your Supporting Information files at the end of your manuscript.

Response: We have added captions for all Supporting Information files at the end of the manuscript (after the References section), in accordance with the journal’s guidelines. The captions are as follows:

“Supplementary Table. Primers used for quantitative reverse transcription PCR (RT-qPCR).

Supplementary Figure 1 Tumors and renal injuries influence the expression of miR-191-5p in serum or urine.”

10. If the reviewer comments include a recommendation to cite specific previously published works, please review and evaluate these publications to determine whether they are relevant and should be cited. There is no requirement to cite these works unless the editor has indicated otherwise.

Response: Not applicable. No citation recommendations were made by the reviewers.

Responds to the reviewer’s comments:

Reviewer #1:

1, the manuscript is not well written. It is full of grammar errors and should be thoroughly checked and corrected by language editor. Even the title is wrongly worded and should cover the fact that all the experiments were conducted in mice.

Response: Thank you for your reminder. We have thoroughly revised the language of the manuscript and hope that the revised version now meets the journal’s standards. We have also revised the title to reflect that all experiments were conducted in mice.

For example:

“The Mechanism of miR-191 Affecting Skeletal Muscle Differentiation by Regulating Wwp1.” was revised to “miR-191 Affects Skeletal Muscle Differentiation by Regulating Wwp1 in Mouse Myoblasts.”

“As crucial cargoes of extracellular vesicles, the regulatory roles of miRNAs in skeletal muscle atrophy associated with COPD, cachexia, and renal injury require further investigation.” was revised to “As crucial cargoes of extracellular vesicles, miRNAs play regulatory roles in skeletal muscle atrophy associated with COPD, cachexia, and renal injury require further investigation.”

2 Second, the materials and methods lack important details but contain irrelevant text that looks like copy/paste from reagent manuals.

Response: Thank you for your careful work. We have added the missing details (transfection medium, reagent, vector, animal numbers, anesthesia/euthanasia, luciferase assay steps, sample sources) and the Materials and Methods has been thoroughly revised.

For example:

“After injection, the mice were placed on a heating table until recovery from anesthesia and subsequently returned to their cages.” was revised to “After injection, the mice were placed on a heating table until recovery from anesthesia and subsequently returned to their cages. Mice were randomly assigned to three groups: PBS‑injected control (n=5, harvested on day 3), CTX‑injected day 1 group (n=3, harvested on day 1), and CTX‑injected day 3 group (n=3, harvested on day 3). At the indicated endpoints, mice were euthanized by cervical dislocation. No analgesics were used to avoid interference with muscle regeneration; mice were monitored twice daily and no severe pain was observed. The animal protocol was approved by the Institutional Animal Care and Use Committee of Inner Mongolia University of Science and Technology (approval No. NMGKJDX‑2022‑9‑22).”

3 miRNA mimics and siRNA should be better characterized and include targeting sites, sequences and catalogue numbers.

Provide the name of the “transfection reagent” used in the study

Provide the name of the “vector” used in the “Transfection protocol for HEK293T Cell”

Response: Thank you for your suggestion.

① We have added detailed information about the miRNA mimics and siRNA in the "Transfection" subsection of the Materials and Methods:

“The sequence of miR-191 mimics is CAACGGAAUCCCAAAAGCAGCUG. The catalog number of NC is miR1N0000002-1-5. The catalog number of siWwp1 is siB12329162409-1-5. The catalog number of siNC is siN0000001-1-5. All of the above nucleic acids were synthesized by RiboBio Co., Ltd. (Guangzhou, China).”

② We have added the name and catalog number of the transfection reagent in the "Transfection" subsection of the Materials and Methods:

“Lipo6000™, C0526.”

③ In the “Transfection protocol for HEK293T Cell” subsection of the Materials and Methods, we have clarified that the vector used is the psiCHECK-2 vector (psi-wt-Wwp1 or psi-m-Wwp1).

The psiCHECK™-2 vector (C8021, Promega) contains dual luciferase reporters (Renilla luciferase and Firefly luciferase) and is used for miRNA target validation. We have described the construction of the vector in the “Construction of psiCHECK-2 Vector” subsection of the Materials and Methods. In this revision, we have added the catalog number of this vector.

4 What medium was used in transfection procedures?

In general, the methods section, specifically transfection part is full of grammar errors, and should be shorten and corrected.

Response: Thank you for your careful work. The medium used during transfection was Opti-MEM™ (51985034, Gibco). We have added this information in the “Transfection” subsection of the Materials and Methods. Meanwhile, we have thoroughly reviewed and revised the Materials and Methods.

For example:

“During this incubation period, remove the old medium from the cells and add 1 mL of fresh serum-free medium.” was revised to “During this incubation period, the old medium was removed from the cells and 1 mL of fresh serum-free medium was added.”

5 How the “the activities of Renilla and Firefly luciferases” was measured?

Response: Thank you for your careful work. We have added the detection process for the activities of Renilla and Firefly luciferases in the “Transfection protocol for HEK293T Cell” subsection, as follows:

“Materials and Methods

Transfection protocol for HEK293T Cell:

Cells were harvested and fully lysed using 1×Passive Lysis Buffer (PLB) from the Dual-Luciferase® kit. Firefly luciferase activity was measured by adding Luciferase Assay Reagent II (LARII), and the luminescence was immediately read using a luminometer. Subsequently, Stop & Glo® Reagent was added to the same well to quench the firefly signal and simultaneously initiate the Renilla luciferase reaction, and the second reading was taken. The ratio of Renilla to Firefly luciferase activity was calculated for each well to normalize for transfection efficiency and cell number. This revised description aligns with standard protocols for the Dual-Luciferase® Reporter Assay System. We believe these additions fully address the reviewer’s concern. Thank you for helping us improve the methodological rigor of our manuscript.”

6 Which “Cells or homogenized tissues” were used in the study?

Response: Thank you for your suggestion. We have revised the RT-qPCR method section to clearly indicate what the cell samples and tissue samples refer to, as follows:

The cell samples refer to the myoblast cell line C2C12 used in the experiments shown in Figures 3–6; the tissue samples refer to the tibialis anterior muscle from the CTX-injected mouse experiment shown in Figures 2C–D.

7 What is the number of animals used in the study?

Response: Thank you for your advice. We thank the reviewer for the question. The number of animals used in this study has been indicated in the Methods section and in the legend of Figure 2C-D, as follows:

For cardiotoxin injection, mice were anesthetized via intraperitoneal administration of pentobarbital sodium (50 mg/kg). The fur overlying the tibialis anterior (TA) muscle was shaved, and the injection site was disinfected with an alcohol swab. Using a microsyringe, 100 μL of CTX solution (10 μM in PBS) was slowly injected into the tibialis anterior muscle. After injection, the mice were placed on a heating table until recovery from anesthesia and subsequently returned to their cages. Mice were randomly assigned to three groups: PBS‑injected control (n=2, harvested on day 1; n=3, harvested on day 3), CTX‑injected day 1 group (n=3, harvested on day 1), and CTX‑injected day 3 group (n=3, harvested on day 3).

8 What had happened to the animals after CTX injection?

Response: Thank you for your attention to animal welfare details. Following unilateral CTX injection into the TA muscle, the injected hindlimb showed transient local inflammation with mild lameness and limited mobility, but the mice could still move and feed on their own. Lameness peaked on day 1, began to improve on day 3, and returned to near-normal gait by day 7. No systemic abnormalities were observed during this period. In our original Materials and Methods, we only described the injection and recovery procedures, but did not provide detailed post-operative observations. The supplementary information is as fo

Attachment Submitted filename: Respond to reviewer miR-191 Regulate Wwp1.docx

# Editor report: Decision Letter 1

**DOI:** 10.1371/journal.pone.0355370.r003

**Authors:** Hongkyun Kim

23 Apr 2026

PONE-D-25-62700R1

miR-191 Affects Skeletal Muscle Differentiation by Regulating Wwp1 in Mouse Myoblasts

PLOS One

Dear Dr. Liu,

Thank you for submitting your manuscript to PLOS ONE. After careful consideration, we feel that it has merit but does not fully meet PLOS ONE’s publication criteria as it currently stands. Therefore, we invite you to submit a revised version of the manuscript that addresses the points raised during the review process.

In my last decision letter, I specifically asked to improve the manuscript by performing the independent verification of altered wwp-1 mRNA levels and mTOR activity. In your cover letter, you have not mentioned any of those concerns. Therefore, I would give you additional time to address my concerns.

Please submit your revised manuscript by Jun 07 2026 11:59PM. If you will need more time than this to complete your revisions, please reply to this message or contact the journal office at plosone@plos.org. When you're ready to submit your revision, log on to https://www.editorialmanager.com/pone/ and select the 'Submissions Needing Revision' folder to locate your manuscript file.

Please include the following items when submitting your revised manuscript:

- A letter that responds to each point raised by the academic editor and reviewer(s). You should upload this letter as a separate file labeled 'Response to Reviewers'.
- A marked-up copy of your manuscript that highlights changes made to the original version. You should upload this as a separate file labeled 'Revised Manuscript with Track Changes'.
- An unmarked version of your revised paper without tracked changes. You should upload this as a separate file labeled 'Manuscript'.

If you would like to make changes to your financial disclosure, please include your updated statement in your cover letter. Guidelines for resubmitting your figure files are available below the reviewer comments at the end of this letter.

If applicable, we recommend that you deposit your laboratory protocols in protocols.io to enhance the reproducibility of your results. Protocols.io assigns your protocol its own identifier (DOI) so that it can be cited independently in the future. For instructions see: https://journals.plos.org/plosone/s/submission-guidelines#loc-laboratory-protocols. Additionally, PLOS ONE offers an option for publishing peer-reviewed Lab Protocol articles, which describe protocols hosted on protocols.io. Read more information on sharing protocols at https://plos.org/protocols?utm_medium=editorial-email&utm_source=authorletters&utm_campaign=protocols.

As the corresponding author, your ORCID iD is verified in the submission system and will appear in the published article. PLOS supports the use of ORCID, and we encourage all coauthors to register for an ORCID iD and use it as well. Please encourage your coauthors to verify their ORCID iD within the submission system before final acceptance, as unverified ORCID iDs will not appear in the published article. Only the individual author can complete the verification step; PLOS staff cannot verify ORCID iDs on behalf of authors.

We look forward to receiving your revised manuscript.

Kind regards,

Hongkyun Kim

Academic Editor

PLOS One

Journal Requirements:

If the reviewer comments include a recommendation to cite specific previously published works, please review and evaluate these publications to determine whether they are relevant and should be cited. There is no requirement to cite these works unless the editor has indicated otherwise.

Additional Editor Comments:

I feel that some conclusions are not really supported by the data. For example, Wwp1 expression is increased in 3 days after CTX injection. However, these data are from RT-qPCR; therefore, it is difficult to conclude whether Wwp1 is increased at the protein level. Moreover, you conclude that miR-191 regulates PI3K/mTOR pathway based on RT-qPCR data. Particularly, the difference in the expression level is a bare minimum with only three samples. Given that there are other options to measure mTOR activity, I recommend that you consider them.

[Note: HTML markup is below. Please do not edit.]

[NOTE: If reviewer comments were submitted as an attachment file, they will be attached to this email and accessible via the submission site. Please log into your account, locate the manuscript record, and check for the action link "View Attachments". If this link does not appear, there are no attachment files.]

To ensure your figures meet our technical requirements, please review our figure guidelines: https://journals.plos.org/plosone/s/figures

You may also use PLOS’s free figure tool, NAAS, to help you prepare publication quality figures: https://journals.plos.org/plosone/s/figures#loc-tools-for-figure-preparation.

NAAS will assess whether your figures meet our technical requirements by comparing each figure against our figure specifications.

# Author comment: Author response to Decision Letter 2

**DOI:** 10.1371/journal.pone.0355370.r004

7 Jun 2026

Dear Editor.

Thank you for your continued guidance and for granting us additional time to revise our manuscript. We have now performed the requested experiments and analyses. Below we address each point point-by-point.

Responds to the editor’s comments:

Comment 1: Please verify altered mTOR activity at the protein level.

Response: Thank you for your suggestion. We have performed Western blot to examine the effect of miR-191 mimics on p-AKT (Ser473) and total mTOR protein levels in C2C12 myoblasts. As shown in the new Figure S2, transfection with miR-191 mimics significantly reduced total mTOR protein expression compared to the negative control. However, no significant change was detected in p-AKT (Ser473) levels. Densitometric analysis was performed using ImageJ. These data indicate that miR-191 downregulates mTOR protein, consistent with its inhibitory effect on Mtor mRNA (Fig 3D). Although p-AKT was not altered, the reduction in mTOR itself may contribute to the observed inhibition of myogenic differentiation. We have added this result to the Results section (lines 349–355) and Supplementary Figure 2.

As follows:

“To further determine whether miR-191 affects the PI3K/mTOR pathway at the protein level, we detected the expression of p-AKT and total mTOR. Western blot analysis revealed that after transfection with miR-191 mimics, total mTOR protein levels were significantly decreased, while p-AKT (Ser473) levels showed no significant change (Fig S2). These findings suggest that miR-191 may be involved in the regulation of myogenic differentiation through downregulation of mTOR protein.”

“Supplementary Figure 2 Western blot analysis of the effect of miR-191 mimics on mTOR and p-AKT protein expression. (A)-(B) miR-191 mimics were transfected before cell differentiation, and differentiation was induced when changing the culture medium after transfection. On day 3 of differentiation, Western blot was used to verify the expression of p-AKT (n=4, shown is a representative result ) and mTOR (n=3). Densitometric analysis was performed using ImageJ. ”

Comment 2: Please verify altered Wwp1 expression at the protein level.

Response: Thank you for your reminder. We fully agree that protein-level evidence would strengthen the conclusion. We tested several commercial anti-Wwp1 antibodies in both C2C12 lysates and mouse skeletal muscle tissues but were unable to obtain specific and reproducible bands under our experimental conditions. We acknowledge this technical limitation. As an alternative validation, we analyzed a published single-nucleus RNA-seq dataset (GSE272412) from mouse tibialis anterior muscle 3 days after CTX injury. By extracting the myocyte subpopulation, we found that in both aged and young mice, the proportion of Wwp1-positive myocytes and the average expression level of Wwp1 within myocytes were significantly increased at day 3 post‑injury (new Fig 2E). This result independently corroborates our qPCR findings (Fig 2D) and supports the upregulation of Wwp1 during skeletal muscle injury/regeneration. We have included this analysis in the Results section and described the approach in Materials and Methods.

As follows:

“Materials and Methods

Analysis of single-nucleus RNA-seq data from a mouse model of CTX-induced skeletal muscle injury (GSE272412)

We analyzed a publicly available single-nucleus RNA-seq dataset (GSE272412), in which mice were subjected to CTX-induced injury in the tibialis anterior muscle and sampled 3 days post-injury. From the expression matrix, we extracted the myocyte subpopulation. For each age group following CTX injury, we calculated (1) the proportion of Wwp1-positive myocytes and (2) the average expression level of Wwp1 within the myocyte population.”

“Results

As an independent validation, we analyzed a published single-nucleus RNA-seq dataset (GSE272412) derived from mouse tibialis anterior muscle at 3 days after CTX injury. By extracting the myocyte subpopulation, we found that in both aged and young mice, the proportion of Wwp1-positive myocytes and the average expression level of Wwp1 within myocytes were significantly increased at 3 days post-CTX injury (Fig. 2E). This result corroborates our qPCR findings (Fig. 2D), further supporting the upregulation of Wwp1 during skeletal muscle injury and regeneration.

(E) Wwp1 expression in myocyte cells. Bubble size indicates the percentage of Wwp1-positive cells; color indicates average expression level.”

Sincerely,

Chuncheng Liu (on behalf of all authors)

Attachment Submitted filename: Respond to reviewer miR-191 Regulate Wwp1 0607.docx

# Peer review: Decision Letter 2

**DOI:** 10.1371/journal.pone.0355370.r005

**Authors:** Hongkyun Kim

21 Jul 2026

miR-191 Affects Skeletal Muscle Differentiation by Regulating Wwp1 in Mouse Myoblasts

PONE-D-25-62700R2

Dear Dr. Liu,

We’re pleased to inform you that your manuscript has been judged scientifically suitable for publication and will be formally accepted for publication once it meets all outstanding technical requirements.

Within one week, you’ll receive an e-mail detailing the required amendments. When these have been addressed, you’ll receive a formal acceptance letter and your manuscript will be scheduled for publication.

An invoice will be generated when your article is formally accepted. Please note, if your institution has a publishing partnership with PLOS and your article meets the relevant criteria, all or part of your publication costs will be covered. Please make sure your user information is up-to-date by logging into Editorial Manager at Editorial Manager® and clicking the ‘Update My Information' link at the top of the page. For questions related to billing, please contact billing support.

If your institution or institutions have a press office, please notify them about your upcoming paper to help maximize its impact. If they’ll be preparing press materials, please inform our press team as soon as possible -- no later than 48 hours after receiving the formal acceptance. Your manuscript will remain under strict press embargo until 2 pm Eastern Time on the date of publication. For more information, please contact onepress@plos.org.

Kind regards,

Hongkyun Kim

Academic Editor

PLOS One

Staff editor comments: We noted that the background may have been altered in all the provided original blot images. It is important that the background of original blot images should not be over adjusted. We ask you resubmit the original blot images.

Reviewers' comments:

Reviewer's Responses to Questions

**Comments to the Author**

Reviewer #1: (No Response)

**********

2. Is the manuscript technically sound, and do the data support the conclusions?

Reviewer #1: (No Response)

**********

3. Has the statistical analysis been performed appropriately and rigorously?

Reviewer #1: (No Response)

**********

4. Have the authors made all data underlying the findings in their manuscript fully available?

The PLOS Data policy

Reviewer #1: (No Response)

**********

5. Is the manuscript presented in an intelligible fashion and written in standard English?

Reviewer #1: (No Response)

**********

Reviewer #1: (No Response)

**********

what does this mean?). If published, this will include your full peer review and any attached files.

If you choose “no”, your identity will remain anonymous but your review may still be made public.

**Do you want your identity to be public for this peer review?**  For information about this choice, including consent withdrawal, please see our Privacy Policy

Reviewer #1: No

**********

# Editor report: Acceptance letter

**DOI:** 10.1371/journal.pone.0355370.r006

**Authors:** Hongkyun Kim

PONE-D-25-62700R2

PLOS One

Dear Dr. Liu,

I'm pleased to inform you that your manuscript has been deemed suitable for publication in PLOS One. Congratulations! Your manuscript is now being handed over to our production team.

At this stage, our production department will prepare your paper for publication. This includes ensuring the following:

* All references, tables, and figures are properly cited

* All relevant supporting information is included in the manuscript submission,

* There are no issues that prevent the paper from being properly typeset

You will receive further instructions from the production team, including instructions on how to review your proof when it is ready. Please keep in mind that we are working through a large volume of accepted articles, so please give us a few days to review your paper and let you know the next and final steps.

Lastly, if your institution or institutions have a press office, please let them know about your upcoming paper now to help maximize its impact. If they'll be preparing press materials, please inform our press team within the next 48 hours. Your manuscript will remain under strict press embargo until 2 pm Eastern Time on the date of publication. For more information, please contact onepress@plos.org.

You will receive an invoice from PLOS for your publication fee after your manuscript has reached the completed accept phase. If you receive an email requesting payment before acceptance or for any other service, this may be a phishing scheme. Learn how to identify phishing emails and protect your accounts at https://explore.plos.org/phishing.

If we can help with anything else, please email us at customercare@plos.org.

Thank you for submitting your work to PLOS One and supporting open access.

Kind regards,

PLOS One Editorial Office Staff

on behalf of

Dr. Hongkyun Kim

Academic Editor

PLOS One
